# Characterization of DNA methylation as well as mico-RNA expression and screening of epigenetic markers in adipogenesis

**DOI:** 10.1186/s12967-022-03295-w

**Published:** 2022-02-15

**Authors:** Yong Zhang, Fancheng Chen, Fangxue Zhang, Xiaowei Huang

**Affiliations:** 1 Department of Orthopedics, the First Affiliated Hospital of Soochow University, Suzhou, China; 2 Department of Orthopaedics, Zhongshan Hospital, Fudan University, Shanghai, China; 3 Knee Surgery Department of the Institute of Sports Medicine, Beijing Key Laboratory of Sports Injuries, Peking University Third Hospital, Peking University, Beijing, China

**Keywords:** Adipogenesis, Methylation, Micro-RNA, Epigenetics

## Abstract

This study aimed to use bioinformatics methods to characterize epigenetic changes in terms of micro-RNA(miRNA) expression and DNA methylation during adipogenesis. The mRNA and miRNA expression microarray and DNA methylation dataset were obtained from the GEO database. Differentially expressed genes (DEGs), differentially expressed miRNAs (DEMs) and differentially methylated probes (DMPs) were filtered using the limma package. The R language cluster profile package was used for functional and enrichment analysis. A protein–protein interaction (PPI) network was constructed using STRING and visualized in Cytoscape. The Connection map (CMap) website tool was used to screen potential therapeutic drugs for adipogenesis. When comparing the early and late stages of adipogenesis, 111 low miRNA targeted upregulated genes and 64 high miRNA targeted downregulated genes were obtained, as well as 663 low-methylated high-expressed genes and 237 high-methylated low-expressed genes. In addition, 41 genes (24 upregulated and 17 downregulated) were simultaneously regulated by abnormal miRNA changes and DNA methylation. Ten chemicals were identified as putative therapeutics for adipogenesis. In addition, among the dual-regulated genes identified, CANX, HNRNPA1, MCL1, and PPIF may play key roles in the epigenetic regulation of adipogenesis and may serve as aberrant methylation or miRNA targeting biomarkers.

**Supplementary Information:**

The online version contains supplementary material available at 10.1186/s12967-022-03295-w.

## Introduction

With the continuous improvement in living standards, obesity has become a worldwide public health problem. Unhealthy lifestyles and eating habits have caused the number of obese people to rapidly increase worldwide. From 2011 to 2012, 8.1% of children aged 0 to 2 in the United States were overweight. The proportion of obese children among children aged 2–19 years reached 16.9%, and the proportion of obesity among adults aged 20 and above reached 34.9% [1]. Obesity is caused by energy intake exceeding energy expenditure. At the cellular level, obesity is the result of an increase in the number or volume of adipocytes [2].

All adipocytes, along with osteoblasts, muscle cells, and chondrocytes, are derived from mesenchymal stem cells. This process of differentiation is called adipogenesis [3]. The formation of mature adipocytes includes two stages, commitment and terminal differentiation [4]. In the commitment stage, mesenchymal stem cells are committed to differentiate to preadipocytes. Preadipocytes can differentiate into adipocytes, but preadipocytes will not spontaneously undergo terminal differentiation without exogenous adipogenesis-stimulating factors [5]. In vitro, when adipogenesis-stimulating factors, including glucocorticoids, cAMP agonists, and insulin, are added, preadipocytes are induced to differentiate into mature adipocytes. In the terminal stage, preadipocytes undergo lipid accumulation and morphological changes of turning spheral. A series of signaling pathways, transcription factors and related proteins are subsequently activated in this process [6].

MicroRNAs (miRNAs) can interact with transcription factors and important signaling molecules related to adipocyte differentiation to regulate adipogenesis [7]. PPARγ and C/EBPs are the most important transcription factors throughout the process of adipocyte differentiation. miRNAs can directly or indirectly interact with these transcription factors to regulate cell differentiation [8]. Kim et al. [9] and Lee et al. [10] found that miR-27a and miR-130a bind with the 3′UTR of PPARγ to downregulate the expression of PPARγ. MiR-27a and miR-130a are downregulated during the differentiation of 3T3-L1 cells, which upregulates the expression of PPARγ. Yang et al. [11] found that the levels of miR-138 were significantly decreased during the adipogenic differentiation of primary adipose stem cells. When miR-138 is overexpressed, adipogenic genes, such as PPARγ, C/EBPα, and FABP4, are inhibited; therefore, lipid droplet aggregation is reduced.

It has also been reported that the DNA methylation of several key genes affects their expression levels during adipogenesis [12]. Leptin is a hormone that regulates energy homeostasis. The leptin promoter is rich in CpG sites and is a chromosomal tissue-specific methyl region that can be dynamically methylated in humans and mice. Detecting the changes in DNA methylation at CpG sites in the promoter region of the leptin gene before and after differentiation of 3T3-L1 cells confirmed that the degree of DNA methylation was reduced, and DNA demethylation promoted leptin gene expression in 3T3-L1 cells [13]. Yokomori et al. [14] found that the GLUT4 promoter region exhibited similar changes in DNA methylation during 3T3-L1 differentiation. Horii et al. [15] screened a small G protein Rho family guanine nucleotide exchange factor (Rho guanine nucleotide exchange factor 19, ARHGEF19) gene using a methylation-sensitive endonuclease PCR method and demonstrated that this gene plays an important role in regulating adipocyte differentiation. Studies on PPARγ, the key regulator of adipocyte differentiation, found that 5-aza-2′-deoxycytidine (AzaD), a DNA methylation inhibitor, interferes with the normal differentiation of 3T3-L1 preadipocytes and inhibits fat accumulation [16].

As aforementioned, the differentiation of preadipocytes into adipocytes is controlled by a regulatory network, which is responsible for the regulation of commitment, lipid accumulation, adipocyte phenotype development, and maturation [17]. The regulatory mechanism can target both stages involved at the same time.

It is worth noting that the expansion of adipose tissue through de novo adipogenesis can neutralize the detrimental metabolic effects secondary to obesity. In addition, the balance of hypertrophy or expansion of existing adipocytes and adipogenesis within the individual has a profound impact on metabolic health. Studies have shown that the increase in adipocyte size was associated with an increased risk of systemic insulin resistance [18]. Other studies have shown that small adipocytes are particularly crucial for suppressing obesity and related metabolic disorders [19] and it was revealed that small adipocytes are usually correlated with reduced susceptibility to diabetes [20]. As their size expands, adipocytes will experience more mechanical stress as their contact pressure with neighboring structures increases, and when they expand to a size close to the oxygen diffusion limit, deficiency of oxygen occurs. The increased mechanical and hypoxic stress of hypertrophic adipocytes can cause inflammation of adipose tissue [21]. Many experimental observations have shown that, compared with smaller adipocytes, larger hypertrophic adipocytes may exhibit different biochemical properties, for example, increased lipolysis, strengthened secretion of inflammatory cytokines, and reduced anti-inflammatory adipokines secretion [22].

These findings raise an intriguing hypothesis that obesity itself may not be responsible for obesity-related metabolic disorders, but the deficiency of adipose tissue to expand further. This can lead to adipocytes hypertrophy instead of hyperplasia and a continuous increase in plasma glucose and lipid levels that can accumulate in other tissues and cause insulin resistance. Based on this theory, promoting de novo adipogenesis or inhibiting adipocyte hypertrophy can be a feasible therapeutic approach for insulin resistance and chronic inflammation secondary to obesity. Consequently, the intervention strategy for adipogenesis should be different between early-stage (promotion) and late-stage (inhibition).

This study systematically analyzed and summarized the epigenetic regulatory network of adipocyte differentiation between the early and late stages through expression profiling, noncoding RNA, and methylation sequencing data derived from public databases. This study aimed to construct a miRNA and DNA methylation regulatory network and to screen out pivotal genes that may provide a more comprehensive understanding of the epigenetic regulation of adipogenesis and potential therapeutic targets in the early and late stages of adipogenesis for the treatment of metabolic diseases.

## Material and methods

### Microarray analysis

The data used in this study were downloaded from the Gene Expression Omnibus (GEO) [23]. GSE59684 was obtained as the noncoding RNA data. DNA methylation data were obtained using the accession number GSE119539. Transcriptome datasets were obtained under the accession numbers GSE76131 and GSE119593. The data are the sequencing data of different stages of mesenchymal stem cells after lipogenic induction. Transcriptome data included sequencing data 0, 6, 48, 96, 192, and 384 h after induction of human SBGS preadipocyte cells. Micro-RNA data included sequencing data from the human MSC line (hMSC-TERT) 0, 7 d, and 13 days after induction. The methylation data are methylation sequencing data from human SBGS preadipocyte cells 0, 24, 48, 96, 192, and 384 h after induction. An induction time of 96 h was set as the cutoff value. Samples with induction times longer than 96 h were classified as a late-stage group, while others were regarded as an early-stage group.

### Analysis of differentially expressed probes

Filtering of differentially expressed probes was accomplished using the R language Bioconductor package. The limma package was used to screen differential expression probes. Absolute t > 2 and q < 0.05 were used as cutoff values. Through the annotation file, the probe names were converted to the gene name, and DEGs and DEMs were then screened out. Differentially methylated CpG probes (DMPs) were filtered similarly. DMPs located in the gene region were well anchored to differentially methylated genes (DMGs). The single methylation value is merged into each gene promoter represented in the GPL13534 platform using the median of the CpG probe methylation value to locate the promoter region, including 1500 nucleotides from the transcription start site TSS1500 and 200 nucleotides from the transcription start site TSS200, 5'UTR and the first exon. The sex chromosome data were not included for analysis.

### PPI network analysis

The human protein interaction pairs were downloaded from the STRING database [23]. Cytoscape software was used to visualize the PPI network based on the output files of the STRING database. The MCODE plugin was then used to identify the pivotal gene modulus with the following parameters: degree cutoff: 2; node score cutoff: 0.2; K-Core: 2; and maximum depth: 100.

### miRNA target gene prediction, miRNA-mRNA network construction, and ceRNA network prediction

The miRWalk3.0 online database was utilized to predict miRNA target genes based on miRNA sequences[24]. The filter parameters were set as score = 1, binding site 3UTR, and experimentally validated. Using the starBase 3.0 database, lncRNAs and circRNAs were predicted based on miRNA sequence input and predicted ceRNA networks were constructed. The parameters were set to CLIP-Data greater than or equal to 1, Degradome-Data greater than or equal to 0, pan-Cancer greater than or equal to 0, and target to empty.

### Functional annotation and pathway enrichment analysis

GO and KEGG enrichment analyses were performed using the R language cluster profile package. The ClueGO plug-in was used to analyze the connections between different GO terms. The R package ggplot2 was employed for visualization.

### Predicting therapeutic drugs based on the CMap database

The query function of the CMap database (https://clue.io/) was used to import upregulated and downregulated gene lists. The exported results are shown in the form of heatmaps. The column is the cell ID, and the row represents perturbation, indicating the classification of the database (small molecule composition, knockout, or overexpression). The small molecule compounds that act on the introduced genes were screened out and ranked in descending order according to the enrichment scores from the heatmap, and the top 10 compounds were selected as potential therapeutic drugs.

### Molecular docking

Maestro (version 10.2) was used to predict the biological binding between compounds and target proteins encoded by hub genes. The protein and chemical structure were imported into Maestro software. After the assignment of the bond sequence, the addition of hydrogen, generation of zero-order bonds with the metal, and generation of disulfide bonds, the preparation was complete, and the structure was ready for docking.

## Results

### Summary of mRNA, miRNA, and CpG islets filtered out

**Fig. 1:**
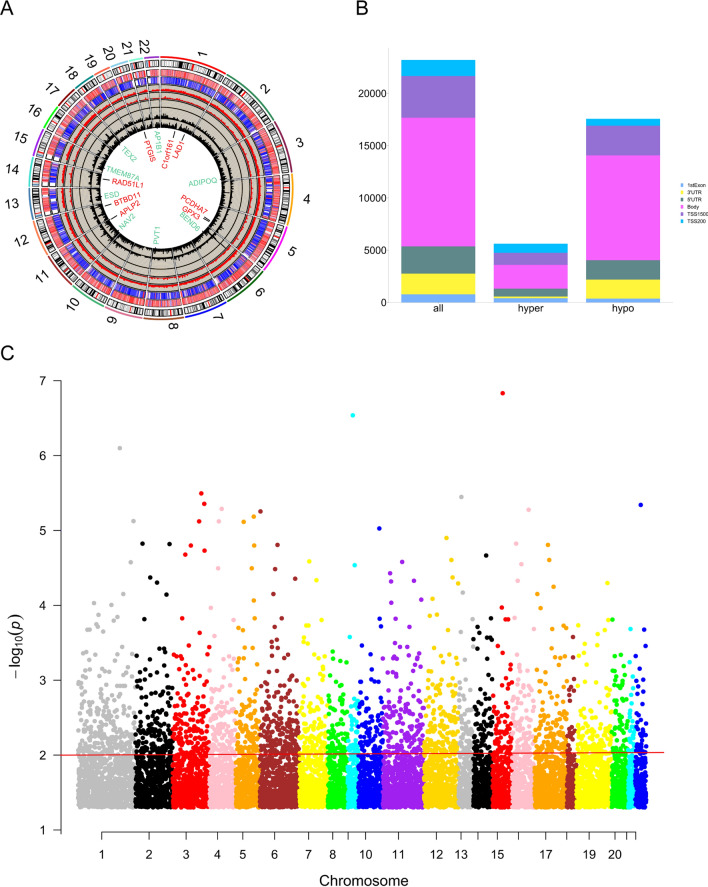
Differential DNA methylation distribution. **A** Circus plot of CpGs. Autosomal chromosomes are shown in a clockwise direction from 1 to 22 in the outermost circle. Red- and green-labeled genes correspond to the top 8 hypermethylated and hypomethylated genes, respectively. The two innermost circles represent the frequency of the filtered hypermethylated (inner) and hypomethylated (outer) CpG islets. The two middle circles show the histogram of p values of hypermethylated (inner) and hypomethylated (outer) CpG islets. The two outermost circles show the heatmaps with two different sample sets of methylated CpG islets. **B** Bar plot of differentially methylated CpGs throughout each genomic region. Body methylated CpG islets were excluded from further analysis. **C** Manhattan plot of epigenome-wide association results showing -log10 (P-value) labeled with the red line

**Fig. 2:**
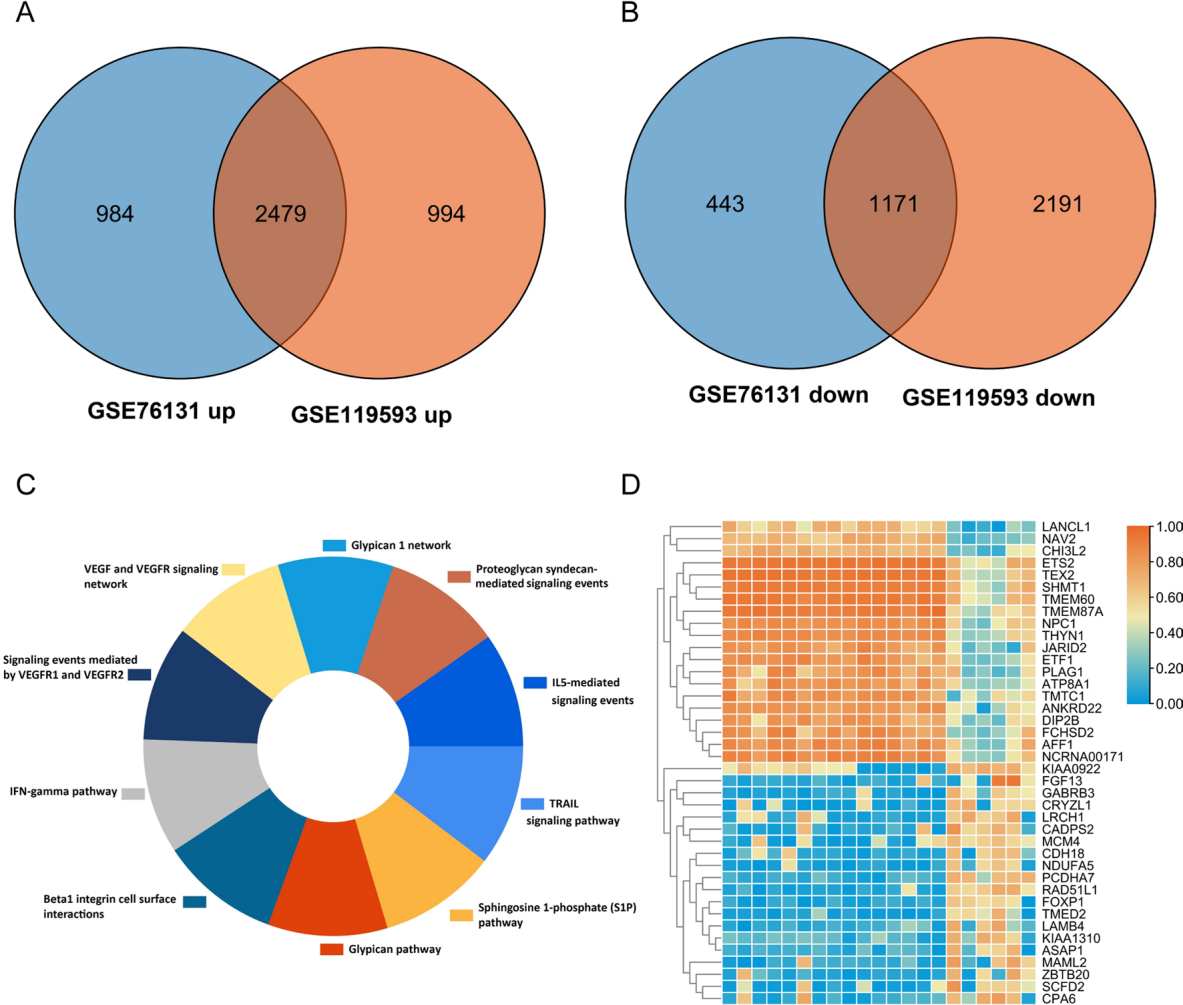
**A** and **B** Venn diagram for all overlapping DEGs, including 2479 upregulated genes and 1171 downregulated genes. **C** KEGG enrichment analysis of DEMs expressed. **D** Top 40 DMGs (20 hypermethylated genes and 20 hypomethylated genes). Orange indicates that the expression of genes is relatively upregulated, and blue indicates that the expression of genes is relatively downregulated

**Fig. 3:**
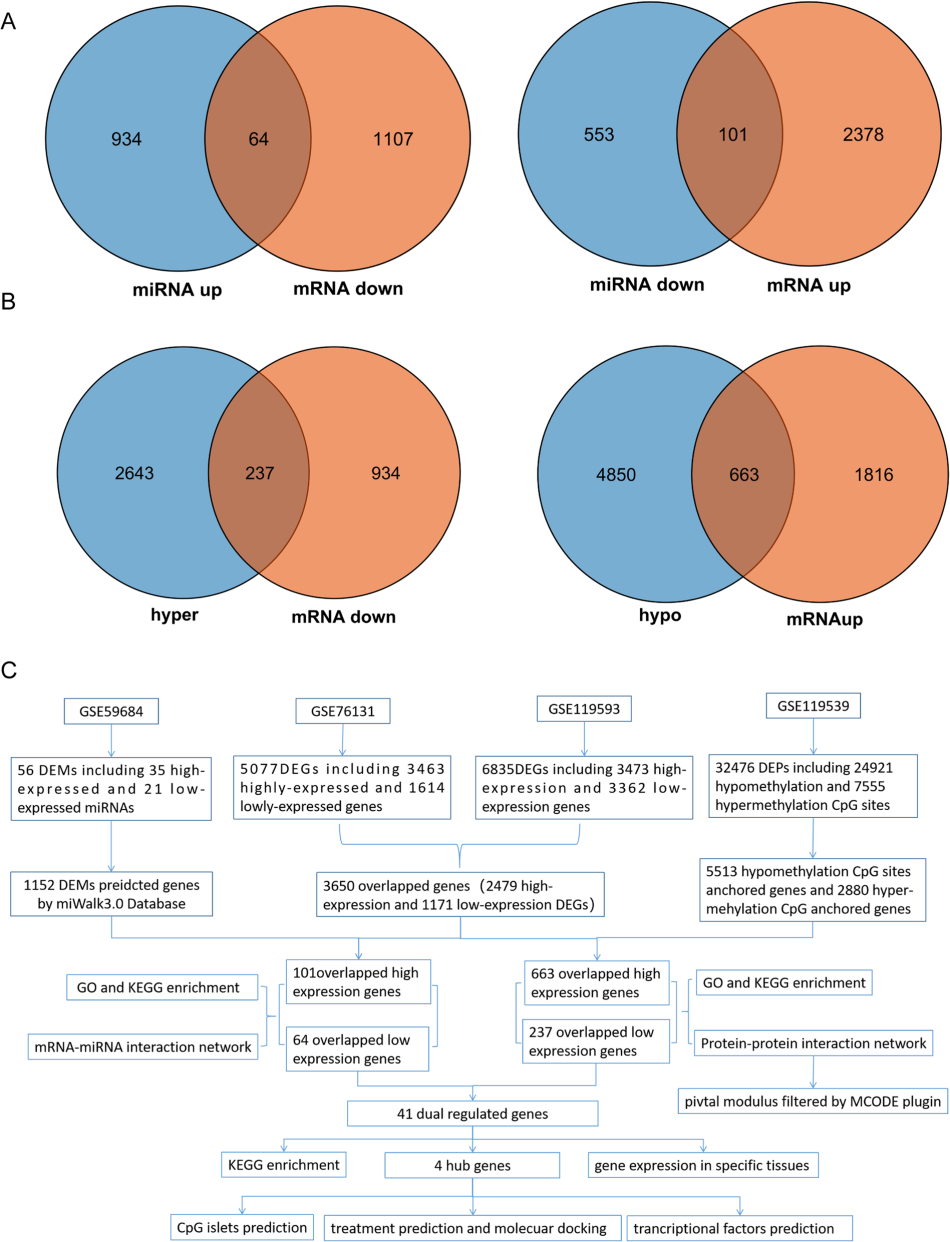
**A** Venn diagram for all overlapping genes between DEM-predicted genes and DEGs. **B** Venn diagram for all overlapping genes between DMGs and DEGs. **C** Overall findings and working flow of the present study

For the gene methylation microarray GSE119539, 24,921 hypomethylated CpG sites were anchored in 5513 genes, and 7,555 hypermethylated CpG sites were anchored in 2880 genes, which were regarded as differentially methylated genes (DMGs). The differentially methylated CpG islets are illustrated in the circus plot (Fig. 1A). Figure 1B shows different methylation site proportions in different genomic subregions. The pattern of DMG distribution on autosomes is shown in Fig. 1C. In GSE76131, a total of 5,077 DEGs were screened out, including 3463 highly expressed and 1,614 lowly expressed genes. In GSE119593, 6835 DEGs were filtered, including 3473 high-expression and 3362 low-expression DEGs. Venn diagrams were utilized to obtain the intersection of the two datasets, and a total of 3650 overlapping DEGs were screened, including 2479 high-expression and 1171 low-expression DEGs, as shown in Fig. 2A-B. In addition, 35 highly expressed miRNAs and 21 downregulated miRNAs were filtered in GSE59684. The GO enrichment analysis of DEMs is shown in Fig. 2C. Figure 2D shows the heatmap of the top 40 DMGs (20 hypermethylated and hypomethylated genes). In addition, 101 low miRNA-targeted upregulated genes and 64 high miRNA-targeted downregulated genes overlapped according to the Venn diagram (Fig. 3A). 663 hypomethylated-highly expressed genes and 237 hypermethylated-low expressed genes were identified by overlapping abnormally methylated and mRNA genes (Fig. 3B). A schematic diagram demonstrating the overall findings and working flow of the study has been plotted in Fig. 3C.

### High-expression genes binding with low-expression miRNAs

**Fig. 4:**
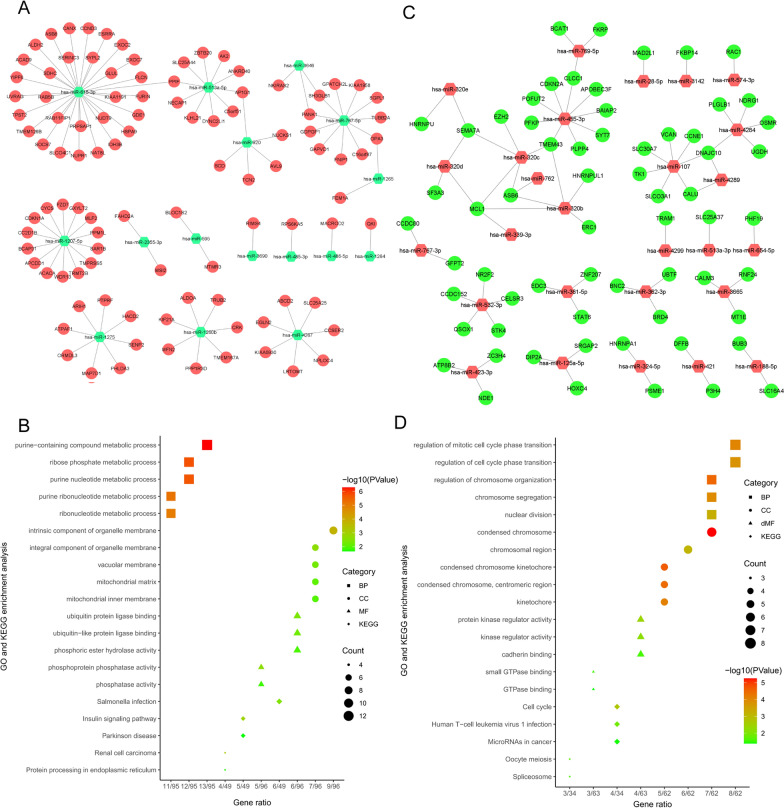
Visualized regulatory network and enrichment bubble graph for miRNA-targeting DEGs. **A** Regulatory network graphs of low-expression miRNAs and upregulated target genes. **B** KEGG and GO enrichment bubble graph for overlapping upregulated genes. **C** Regulatory network graph of highly expressed miRNAs and downregulated genes. **D** KEGG and GO enrichment bubble graph for overlapping downregulated genes

Thirty-two gene ontology terms met the threshold of q value < 0.05. These genes were significantly enriched in nucleotide metabolism and phosphatase activities. To further determine the importance of miRNA regulation in the adipogenesis process, a miRNA-mRNA regulation network was built (Fig. 4A). AP1G1, OPA3, PANK1, PPIF, and SH3GLB1 could be targeted by two miRNAs based on the regulatory network. The bubble chart of KEGG enrichment is shown in Fig. 4B.

### Low-expression genes with high-expression miRNAs

The GO enrichment results showed that genes with low expression overlapping with highly expressed miRNAs were primarily related to cell cycle regulation and chromosome regulation. As plotted in the miRNA-mRNA network, MCL1 was regulated by 4 miRNAs, and ASB6, DNAJC10, SEMA7A, and TMEM43 were regulated by 3 miRNAs. CALU and CLCC1 were regulated by 2 miRNAs, as shown in Fig. 4C. The terms for enriched KEGG analysis are shown as bubble plots in Fig. 4D.

### Highly expressed and hypomethylated genes

**Fig. 5:**
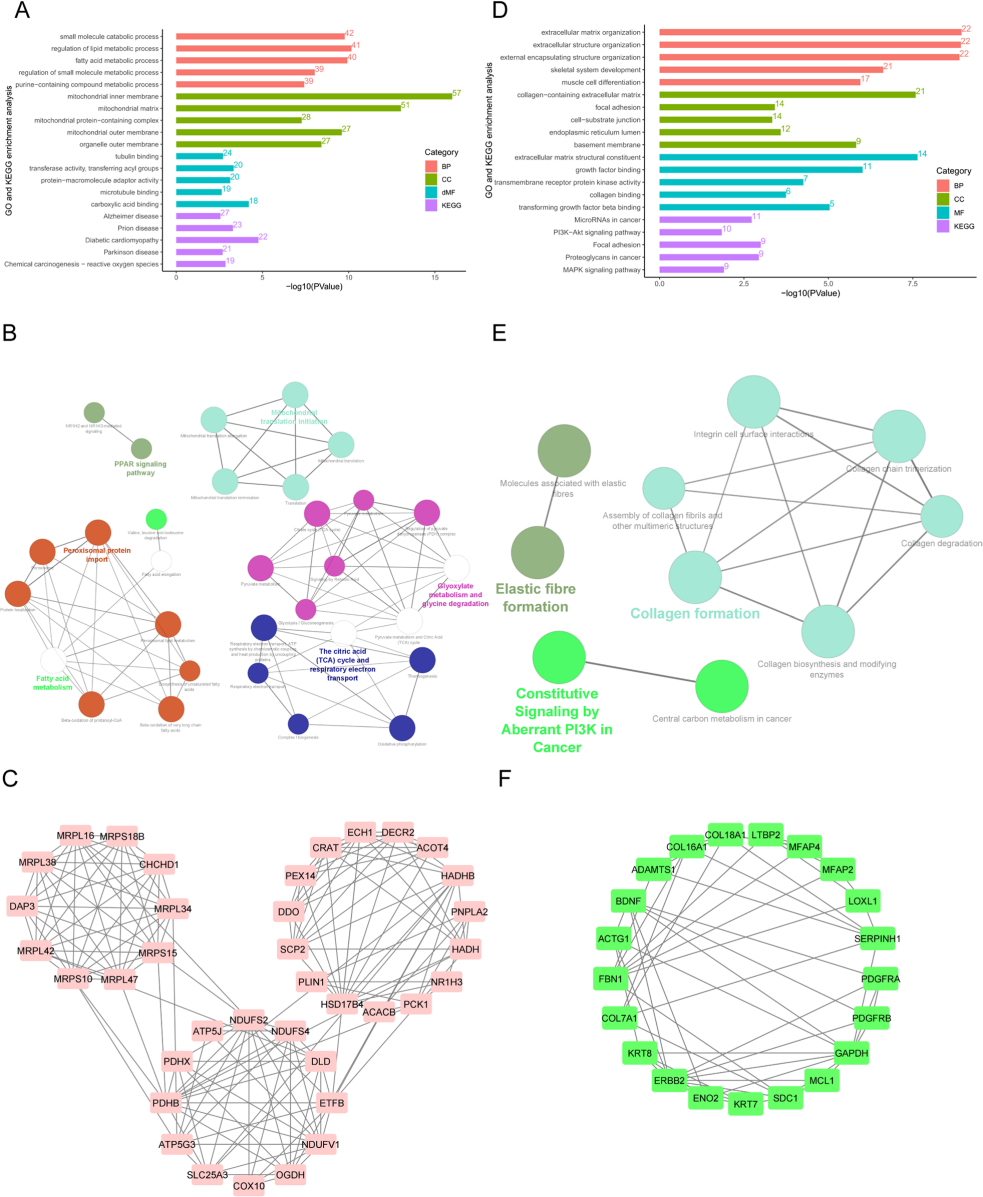
The PPI network and enrichment bar graph for methylation-related DEGs. **A** and **D** GO and KEGG enrichment bar graphs for hypomethylation–upregulated genes (**A**) and hypermethylation–downregulated genes (**D**). **B** and **E** Reactome and KEGG pathway enrichment visualized by CluoGO of hypomethylation and high-expression hub genes (**B**) and hypermethylation and low-expression hub genes (**E**). **C** and **F**, Pivotal modules of hypomethylated–upregulated genes (**C**) and hypermethylated–downregulated genes (**F**)

Enrichment analysis was performed on 364 hypomethylated and highly expressed genes. Among the 335 GO enrichment items filtered using the threshold value of q value < 0.05, the top 5 items were primarily related to lipid metabolism and small molecule catabolism (Fig. 5A). KEGG and Reactome pathway analysis showed that the module was primarily related to the PPAR signaling pathway, fatty acid metabolism, and mitochondrial translation initiation, as illustrated in Fig. 5B. The PPI included 577 genes and established 4274 interconnections using the STRING database. In addition, the MCODE plug-in was used to screen out the most important module based on the generated PPI network (Fig. 5C).

### Downregulated DGEs overlapped with hypermethylation

*A total of* 112 GO terms were screened according to the threshold of q < 0.05. Figure 5D shows that they were primarily involved in the extracellular matrix organization, focal adhesion, and collagen binding. GO and Reactome pathways showed that the function of the core module was primarily related to elastic fiber formation, collagen formation, and constitutive signaling by aberrant PI3K in the cancer pathway, as shown in Fig. 5E. A PPI comprising 173 genes and 744 connections was built using the STRING database. The MCODE plug-in was used to filter out the most significant module (Fig. 5F).

### Dual regulated DEGs

**Fig. 6:**
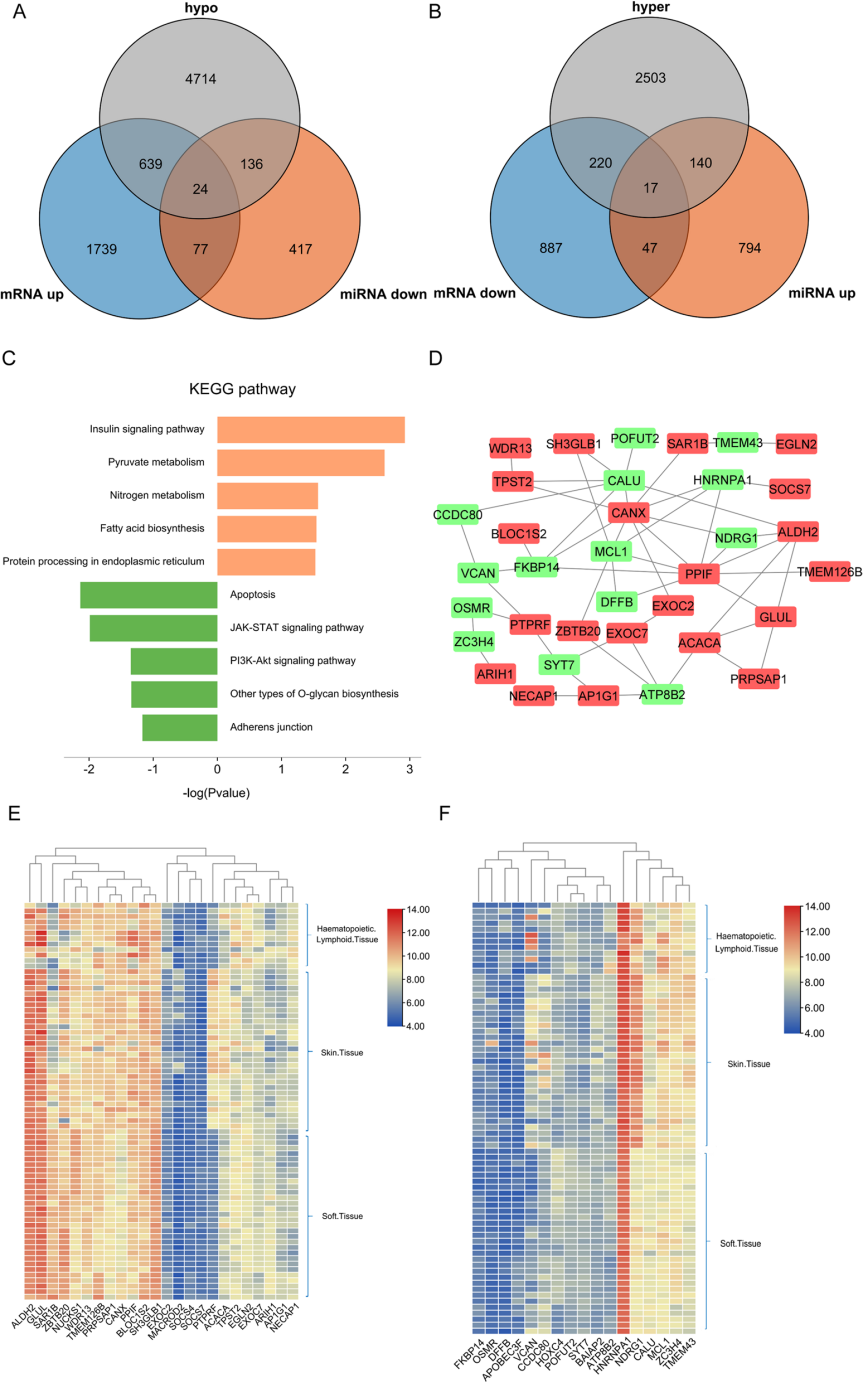
Details of dual regulated genes. **A** and **B**, Venn graph for all overlapping genes, including 17 upregulated genes and 24 downregulated genes. **C** KEGG enrichment analysis of dual regulated genes. Orange indicates the up-regulated genes, and green color indicates the down-regulated genes. **D** PPI network of the overlapping dual regulated genes, including an upregulated label with red color and downregulated genes with green color. **E** and **F** Heatmap of 43 upregulated and 14 downregulated genes expressed in different tissues

Interestingly, some DEGs were simultaneously regulated by both DNA methylation and miRNA, which may indicate a more intricate function during adipogenic differentiation. Twenty-four DEGs, including ACACA, ALDH2, AP1G1, and ARIH1, were upregulated during hypomethylation and low miRNA expression (Fig. 6A). Seventeen genes, including BAIAP2, CALU, CCDC80, and DFFB, were downregulated during hypermethylation and high miRNA expression (Fig. 6B). Additional file 1: Table S3 summarizes DNA methylation sites and their relationship with CpG islands, as well as regulatory miRNAs. The KEGG pathways of up-and down-regulated genes were listed in Fig. 6C. The PPI network of 41 dual-regulated genes is shown in Fig. 6D. To gain deeper insights into the upregulation and downregulation of gene expression across the entire human genome, especially in the hematopoietic system and soft tissues where there exist abundant adipocytes, the MERAV online database was used, and representative heatmaps were created. Among the 24 upregulated genes, ALDH2 and GLUL exhibited the highest expression levels in the hematopoietic system, skin, and soft tissues, as shown in Fig. 6E. Among the 17 downregulated genes, FKBP14, OSMR, and DFFB displayed the lowest expression levels in the hematopoietic system, skin, and soft tissues, as illustrated in Fig. 6F.

Twenty-four up- and 17 downregulated dual overlapping genes were imported to the CMap tool to predict potential compounds that have pharmacologic actions against the input genes. Based on enrichment scores, the top 10 compounds were determined as potential therapeutic candidates for adipogenesis-related pathological conditions, which are listed in Table 1.

**Table1:** Top 10 chemicals were predicted as putative therapeutic agents for adipogenesis

Chemical name	Function	Chemical formula	Enrichmentscore
dolasetron	Serotonin receptor antagonist		1.99
dopamine	Dopamine receptor agonist		1.87
everolimus	MTOR inhibitor		1.86
ivermectin	GABA receptor agonist		1.83
atenolol	Adrenergic receptor antagonist		1.81
dephostatin	Tyrosine phosphatase inhibitor		1.81
nikkomycin	Chitin inhibitor		1.77
roscovitine	CDK inhibitor		1.76
UK-356618	Metalloproteinase inhibitor		1.75
GR-144053	Integrin inhibitor		1.74

**Fig. 7:**
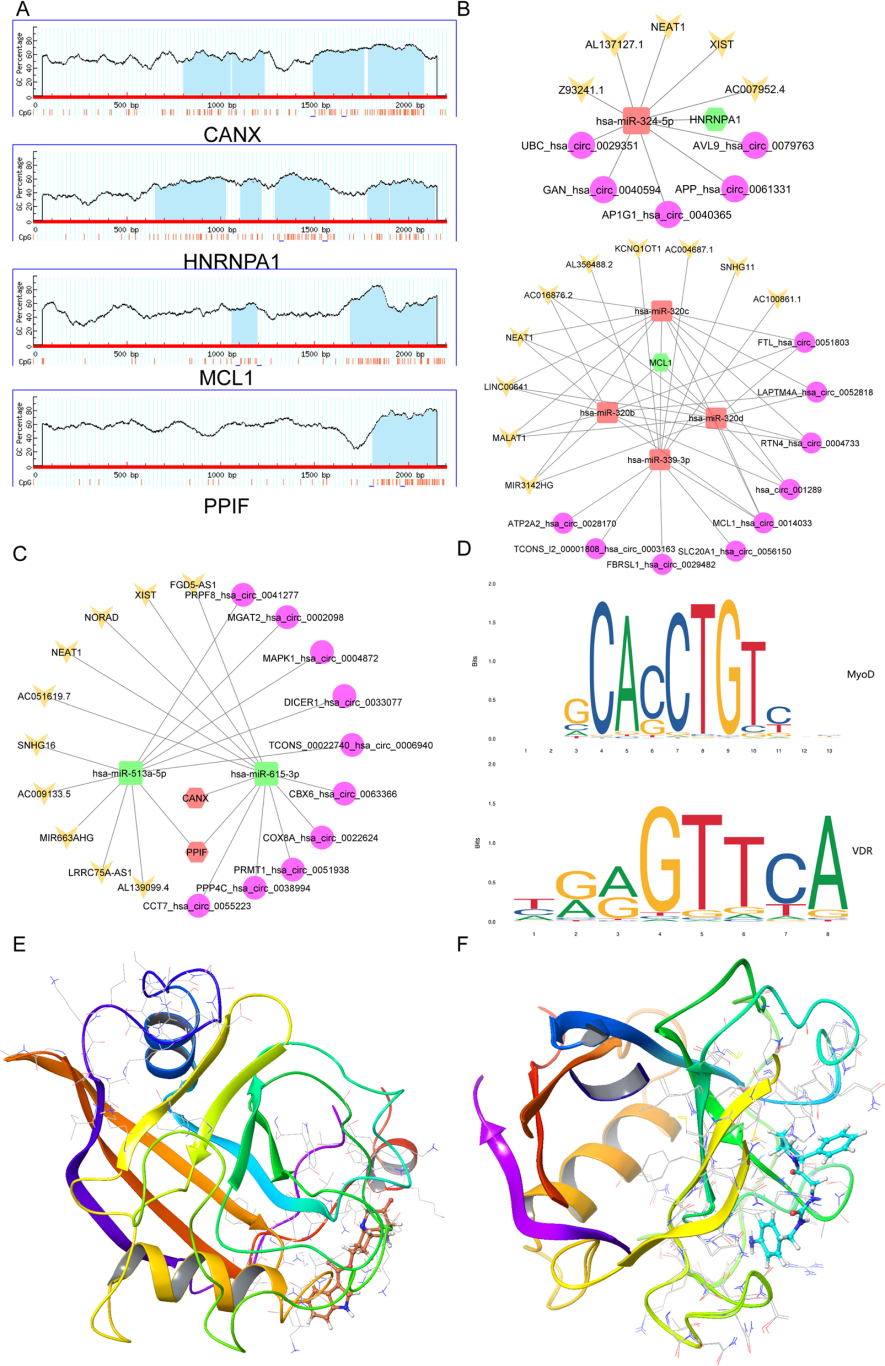
Comprehensive epigenetic regulation prediction of the four hub genes. **A** CpG islands prediction analysis of 4 hub genes. **B** and **C** Ce-RNA regulatory network of four hub genes. **D** Representative predicted transcription factors MyoD and VDR that can bind CpG islands in the promoter regions of CANX. **E** and **F** simulation of the molecular docking pattern of CANX (**E**) and PPIF (**F**) with dolasetron

The predicted CpG island location that can be bound by transcription factors is listed in Fig. 7A. In addition, PPI networks were built based on overlapping abnormally expressed genes, and the core module was screened out using the MCOD plug-in. The core module included four genes, among which the upregulated genes were CANX and PPIF, and the downregulated genes were MCL1 and HNRNPA1. Figures 7B-C show the predicted ceRNA regulation patterns for the four selected genes. In addition, PROMO software has been used to predict the transcription factors that can bind to the methylated regions of the above four gene promoters (Fig. 7D and Additional file 1: Figure S1). To determine the binding pattern between hub gene-encoded proteins and predicted compounds, docking analysis was performed. The docking patterns of CANX and PPIF are shown in Figs. 7E and 7F, respectively. However, further experiments are needed to verify these associations.

## Discussion

The formation of mature adipocytes includes two stages, commitment and terminal differentiation. Once differentiation is initiated, preadipocytes will first enter the contact inhibition phase. When the adipogenesis stimulating factor is added, the preadipocyte morphology will enter the second phase. With the accumulation of lipid droplets, they will transform into mature adipocytes [25]. This article primarily examined the process of adipocyte differentiation in molecular mechanisms and epigenetic regulation during adipogenesis. The miRNA microarray, DNA methylation microarray, and mRNA microarray data were systematically analyzed, and analyses between samples in the early and late stages of adipogenic differentiation were compared. Hub genes and key pathways involved in epigenetic regulation during adipogenic differentiation were screened out.

In this study, day 4 (96 h) of induction was regarded as the cutoff, based on which the cell cohorts were divided into early-stage and late-stage groups. For miRNA datasets, there were no significant probes screened out between days 7 and 13. In addition, for DNA methylation datasets, when 48 h was selected as the cutoff, only 33 significantly methylated CpG islets were screened out. Combined with the aforementioned information, 96 h was selected as the optimal cutoff value.

Through the overlapping strategy of DEG and DEM target genes, 101 upregulated genes with low miRNAs were screened out. KEGG analysis revealed that the insulin signaling pathway was enriched. The insulin signaling pathway has been widely investigated in fat metabolism [26]. After insulin binds to the extracellular alpha subunit of the insulin receptor (InsR), it activates tyrosine kinase activity of the intracellular beta subunit, and the beta subunit undergoes autophosphorylation so that the tyrosine of the downstream substrate is phosphorylated and activated [27]. Insulin promotes the synthesis of fat and glycogen by acting on target tissues and inhibits the decomposition rate of lipids and liver glycogen, strongly promoting the storage of substances in the body. If insulin resistance or insulin production defects occur in the body, it will lead to abnormal regulation of adipogenesis [28].

Sixty-four lowly expressed genes binding to miRNAs with high expression were filtered by determining the intersection between DEGs and DEM target genes. Using GO analysis, it was found that these genes are primarily involved in cell cycle regulation and chromosome regulation. KEGG analysis revealed that these genes were primarily responsible for the cell cycle and proliferation. As illustrated in Fig. 4C of the miRNA-mRNA network, it is worth noting that a total of 7 genes were bound by multiple miRNAs, which may be key targets for the regulation of multiple miRNAs. For instance, hsa-miR-455-3p can bind abundant target genes, such as APOBEC3F, BAIAP2, CDKN2A, CLCC1, PFKP, PLPP4, POFUT2, SYT7, and TMEM43. Hsa-miR-455 enhances adipogenic differentiation of 3T3-L1 cells by targeting uncoupling protein-1 [29]. Hsa-miR-455 activates AMPKα1 by targeting HIF1, and AMPK promotes the brown adipogenic program and mitochondrial biogenesis. Concomitantly, miR-455 also targets the adipogenic suppressors Runx1t1 and Necdin, initiating adipogenic differentiation [30]. KEGG analysis of Hsa-miR-455-3p binding genes revealed that these genes were related to cell cycle regulation and CDC42-related signaling pathways. Recent in vitro experiments confirmed that Cdc42 promotes the differentiation of adipose-derived mesenchymal stem cells (ADSCs) into pancreatic β-like cells through the Wnt/β-catenin pathway [31].

The pathway enrichment of 663 genes with low methylation and high expression was primarily concentrated in Alzheimer's disease, prion disease, diabetic cardiomyopathy, Parkinson's disease, and reactive oxygen species-related chemical carcinogenesis. Diabetic cardiomyopathy is one of the complications of T2DM. These cellular changes include enhanced adipogenesis of MSCs, as observed in both type 1 and 2 models of diabetes. Emerging evidence now implicates enhanced marrow adipogenesis and changes to cellular makeup of the marrow in a novel mechanistic link between various secondary complications of diabetes [32]. The MCODE plug-in was used to screen the core module. Analysis of the core module showed that its function was primarily related to fatty acid metabolism, peroxisomal protein import, the PPAR signaling pathway, the citric acid cycle, respiratory electron transport, glyoxylate metabolism and glycine degradation, and mitochondrial transition initiation.

For 237 genes with high methylation and low expression, GO and KEGG enrichment analysis indicated that they were primarily enriched in the extracellular matrix organization, focal adhesion, and cell-substrate junctions. These genes are primarily related to the movement of cells and formation of the extracellular matrix. The core modules of hypermethylated-low expressed genes were screened out from the PPI network. The functions of the core modules were focused on elastic fiber formation, collagen formation, and constitutive signaling by aberrant PI3K. It was reported that type III collagen (ColIII) is required for 3T3-L1 preadipocyte adipogenesis as well as the formation of actin stress fibers [33]. The above analysis suggests that during the late stage of adipogenesis, cytoskeletal components are reduced, which may be related to the activation of PI3K-related pathways.

Interestingly, miRNA and DNA methylation may work together to regulate the expression of certain genes in adipogenesis. Twenty-four genes, including ACACA, ALDH2, AP1G1, and ARIH1, were increased due to two types of epigenetic regulation. Under dual regulation, 17 genes, such as APOBEC3F, ATP8B2, BAIAP2, and CALU, were downregulated. These genes were primarily involved in apoptosis, the JAK-STAT signaling pathway, the PI3K-Akt signaling pathway, another type of O-glycan biosynthesis, and adherens junctions. For 24 genes upregulated by low miRNA and hypomethylation, KEGG analysis determined that these genes may be involved in the insulin signaling pathway, pyruvate metabolism, nitrogen metabolism, fatty acid biosynthesis, and protein processing in the endoplasmic reticulum. ALDH2 and GLUL were upregulated in hematopoietic and lymphoid tissue and soft tissue. On the other hand, FKBP14, OSMR, and DFFB were downregulated in hematopoietic and lymphoid tissue and soft tissue, indicating that they may be involved in the adipogenesis of fat cells in these tissues.

From the CMap database, 10 chemical substances were identified, including dolasetron, dopamine, everolimus, ivermectin, and atenolol, which may have potential pharmacological actions on adipogenesis. Dopamine is a substance with a wide range of effects, and studies have shown that it has a potential effect on adipogenic differentiation. Dopamine receptor domain 5 (Drd5) genes were previously suggested to contribute to the adipogenesis. Knockdown of dopamine receptor D2 upregulates the expression of adipogenic genes in mouse primary mesencephalic neurons [34].

The MCODE plug-in was used to screen out core modules, including 4 central genes, CANX, HNRNPA1, MCL1, and PPIF. The promoter region of CANX predicts four CpG islands, which may bind a large number of transcription factors, including AP2, MyoD, and VDR. The encoded protein calnexin is a calcium-binding, endoplasmic reticulum (ER)-related protein that transiently interacts with newly synthesized N-linked glycoproteins to promote protein folding and assembly. By keeping misfolded protein subunits in the ER for degradation, it may also play a central role in the quality control of protein folding [35]. HNRNPA1 contains five CpGs in its promoter region. This gene encodes members of the ubiquitously expressed heterogeneous ribonucleoprotein (hnRNP) family. These ribonucleoproteins are RNA-binding proteins associated with pre-mRNA in the nucleus that affects pre-mRNA processing, as well as mRNA metabolism and transport. The protein encoded by this gene is one of the most abundant core proteins of the hnRNP complex and plays a key role in the regulation of alternative splicing. Mutations in this gene have been observed in individuals with amyotrophic lateral sclerosis [36]. MCL1 encodes an anti-apoptotic protein that is a member of the Bcl-2 family. The longest gene product (isotype 1) enhances cell survival by inhibiting apoptosis, while the shorter gene products (isotypes 2 and 3) that are alternately spliced promote apoptosis [37, 38]. The protein encoded by PPIF is a member of the peptidyl-prolyl cis–trans isomerase (PPIase) family. PPIases catalyze the cis–trans isomerization of proline amide peptide bonds in oligopeptides and accelerate protein folding. This protein is part of the mitochondrial permeability transition pore in the inner mitochondrial membrane. Activation of this pore is thought to be related to the induction of apoptosis and necrotic cell death [39].

The role of ceRNA in adipogenesis has been a research hotspot in recent years. For example, lncRNA H19 targets LCoR by interacting with the miR-188 sponge, thereby affecting the osteogenic and adipogenic differentiation process of mouse BMSCs. LncRNA ADNCR inhibits adipogenesis and differentiation by targeting miR-204 [40]. Therefore, potential ceRNA networks have been predicted for the above four central genes.

In the past, it was widely assumed that inhibiting adipogenesis could be a potential anti-obesity approach. However, results from various experiments indicate that adipogenesis inhibitors are not a good choice for improving metabolic disorders because restricting adipocyte expansion may lead to insulin resistance. As reported by Danfour et al., the failure of adipocyte differentiation may lead to type 2 diabetes [17]. The mouse model showed that the improvement of the metabolic health of obese animals can be induced by further healthy expansion of adipose tissue [41]. In these mice, adipogenesis allows adipose tissue to expand in the way of hyperplasia while preventing hypoxia, chronic inflammation, and fibrosis caused by hypertrophy of adipocytes [42]. In the present study, through filtering the pivotal genes and characterizing different epigenetic regulation between the early and late stage of adipogenesis, more precise therapeutic targets could be provided to intervene in the early stage ( promoting hyperplasia) or late stage of adipogenesis (inhibiting hypertrophy) for the treatment of metabolic disorders secondary to obesity. Besides, the genes differentially expressed in the early and late stages of adipogenesis could also be the potential biomarkers to evaluate the risk of metabolic disorders for the overweight population.

There is some inherent limitation in our study. Due to data availability, the study did not include the correlation analysis involving clinical parameters such as incidence rate of metabolic disorders. Besides, more experiments are needed to further validate the effect of specific methylation or miRNA expression changes on adipogenesis. As a consequence, in the future, clinical trials and experiments are planned to validate the effectiveness of the epigenetic regulation of these genes and to explore the feasibility to use certain biomarkers to predict the risk of metabolic disorders among overweight populations.

In summary, this study comprehensively analyzed abnormally methylated, miRNA-targeted, and differentially expressed genes involved in the process of adipogenesis that is epigenetically regulated. Twenty-four genes were upregulated in response to miRNA reduction and hypomethylation, while 17 genes were downregulated in response to miRNA increase and hypermethylation. Ten chemicals were identified as putative therapeutics for adipogenesis. In addition, among these dual-regulated genes, CANX, HNRNPA1, MCL1, and PPIF may be key biomarkers in the epigenetic regulation of adipogenesis and may serve as aberrant methylation or miRNA targeting biomarkers.

## Supplementary Information

**Additional file 1: Table S1**. Gene ontology and KEGG pathway analysis of DEGs associated with aberrant miRNA between early stage and late stage samples. **Table S2**. Gene ontology and KEGG pathway analysis of DEGs associated with aberrant DNA methylation between early stage and late stage samples. **Table S3**. DEGs associated with both specific miRNA and DNA methylation CpG sites between early and late stage. **Figure S1**. Transcription factors predicted for hub genes HNRNPA1,PPIF and MCL1 (2 transcription factors per gene.

## Data Availability

The authors declare that the data supporting the findings of this study are available within the article.

## Abbreviations

GEO: Gene Expression Omnibus database
DEGs: Differentially expressed genes
DEMs: Differentially expressed miRNAs
DMGs: Differentially methylated genes
PPI: Protein–protein interaction
CMap: Connection map
SBGS: Simpson Golabi Behmel Syndrome
DMPs: Deferentially methylated CpG probes
STRING database: Search tool for recurring instances of neighbouring genes
MCODE plugin: Molecular Complex Detection
GO: Gene Ontology
KEGG: Kyoto Encyclopedia of Genes and Genomes
MERAV online database: Metabolic gene rapid visualizer online database
ADSCs: Adipose-derived mesenchymal stem cells (ADSCs)
CANX: Calnexin
HNRNPA1: Heterogeneous nuclear ribonucleoprotein A1
MCL1: MCL1 apoptosis regulator
PPIF: Peptidylprolyl isomerase F
ceRNA: Competing endogenous RNAs
ACACA: Acetyl-CoA carboxylase alpha
ALDH2: Aldehyde dehydrogenase 2 family member
AP1G1: Adaptor related protein complex 1 subunit gamma 1
ARIH1: Ariadne RBR E3 ubiquitin protein ligase 1
UTR: Untranslated Regions
ASB6: Ankyrin repeat and SOCS box containing 6
DNAJC10: DnaJ heat shock protein family (Hsp40) member C10
SEMA7A: Semaphorin 7A
TMEM43: Transmembrane protein 43
CALU: Calumenin
CLCC1: Chloride channel CLIC like 1
BAIAP2: BAR/IMD domain containing adaptor protein 2
CCDC80: Coiled-coil domain containing 80
DFFB: DNA fragmentation factor subunit beta
GLUL: Glutamate-ammonia ligase
FKBP14: FKBP prolyl isomerase 14
OSMR: Oncostatin M receptor
APOBEC3F: Apolipoprotein B mRNA editing enzyme catalytic subunit 3F
CDKN2A: Cyclin dependent kinase inhibitor 2A
PFKP: Phosphofructokinase, platelet
PLPP4: Phospholipid phosphatase 4
POFUT2: Protein O-fucosyltransferase 2
SYT7: Synaptotagmin 7
TMEM43: Transmembrane protein 43
GPR56: Adhesion G protein-coupled receptor G1
APOBEC3F: Apolipoprotein B mRNA editing enzyme catalytic subunit 3F
ATP8B2: ATPase phospholipid transporting 8B2
AP2: Cyclase associated actin cytoskeleton regulatory protein 2
MyoD: Myogenic differentiation 1
VDR: Vitamin D receptor

## References

[1] Ogden CL (2014). Prevalence of childhood and adult obesity in the United States, 2011–2012. JAMA.

[2] Ginsberg-Fellner F, Knittle JL (1981). Weight reduction in young obese children. I. Effects on adipose tissue cellularity and metabolism. Pediatr Res.

[3] Musri MM, Gomis R, Parrizas M (2007). Chromatin and chromatin-modifying proteins in adipogenesis. Biochem Cell Biol.

[4] Al-Mansoori L (2021). Role of inflammatory cytokines, growth factors and adipokines in adipogenesis and insulin resistance. Inflammation.

[5] Buttitta LA, Edgar BA (2007). Mechanisms controlling cell cycle exit upon terminal differentiation. Curr Opin Cell Biol.

[6] Ruijtenberg S, van den Heuvel S (2016). Coordinating cell proliferation and differentiation: antagonism between cell cycle regulators and cell type-specific gene expression. Cell Cycle.

[7] Cristancho AG, Lazar MA (2011). Forming functional fat: a growing understanding of adipocyte differentiation. Nat Rev Mol Cell Biol.

[8] Zhang YL (2021). Roles of MicroRNAs in osteogenesis or adipogenesis differentiation of bone marrow stromal progenitor cells. Int J Mol Sci.

[9] Kim SY (2010). miR-27a is a negative regulator of adipocyte differentiation via suppressing PPARgamma expression. Biochem Biophys Res Commun.

[10] Lee EK (2011). miR-130 suppresses adipogenesis by inhibiting peroxisome proliferator-activated receptor gamma expression. Mol Cell Biol.

[11] Yang Z (2011). MicroRNA hsa-miR-138 inhibits adipogenic differentiation of human adipose tissue-derived mesenchymal stem cells through adenovirus EID-1. Stem Cells Dev.

[12] Kubota Y (2021). Epigenetic modifications underlie the differential adipogenic potential of preadipocytes derived from human subcutaneous fat tissue. Am J Physiol Cell Physiol.

[13] Kuroda M (2016). DNA Methylation Suppresses Leptin Gene in 3T3-L1 Adipocytes. PLoS ONE.

[14] Yokomori N, Tawata M, Onaya T (1999). DNA demethylation during the differentiation of 3T3-L1 cells affects the expression of the mouse GLUT4 gene. Diabetes.

[15] Horii T (2009). Epigenetic regulation of adipocyte differentiation by a Rho guanine nucleotide exchange factor, WGEF. PLoS ONE.

[16] Sakamoto H (2008). Sequential changes in genome-wide DNA methylation status during adipocyte differentiation. Biochem Biophys Res Commun.

[17] Danforth E (2000). Failure of adipocyte differentiation causes type II diabetes mellitus?. Nat Genet.

[18] Salans LB, Knittle JL, Hirsch J (1968). The role of adipose cell size and adipose tissue insulin sensitivity in the carbohydrate intolerance of human obesity. J Clin Invest.

[19] McLaughlin T (2007). Enhanced proportion of small adipose cells in insulin-resistant vs insulin-sensitive obese individuals implicates impaired adipogenesis. Diabetologia.

[20] Lundgren M (2007). Fat cell enlargement is an independent marker of insulin resistance and 'hyperleptinaemia'. Diabetologia.

[21] Shao M (2018). De novo adipocyte differentiation from Pdgfrbeta(+) preadipocytes protects against pathologic visceral adipose expansion in obesity. Nat Commun.

[22] Morley TS, Xia JY, Scherer PE (2015). Selective enhancement of insulin sensitivity in the mature adipocyte is sufficient for systemic metabolic improvements. Nat Commun.

[23] Chen J (2018). Non-small-cell lung cancer pathological subtype-related gene selection and bioinformatics analysis based on gene expression profiles. Mol Clin Oncol.

[24] Zhu W (2020). Epigenetic alternations of microRNAs and DNA methylation contribute to gestational diabetes mellitus. J Cell Mol Med.

[25] Annie-Mathew AS (2021). The pivotal role of Nrf2 activators in adipocyte biology. Pharmacol Res.

[26] Huang S (2021). The blooming intersection of subfatin and metabolic syndrome. Rev Cardiovasc Med.

[27] Salminen A, Kaarniranta K, Kauppinen A (2021). Insulin/IGF-1 signaling promotes immunosuppression via the STAT3 pathway: impact on the aging process and age-related diseases. Inflamm Res.

[28] Khalid M (2021). Insulin signal transduction perturbations in insulin resistance. Int J Mol Sci.

[29] Aguilar-Recarte D (2021). The PPARbeta/delta-AMPK connection in the treatment of insulin resistance. Int J Mol Sci.

[30] Cai Z (2016). MiR-455 enhances adipogenic differentiation of 3T3-L1 cells through targeting uncoupling protein-1. Pharmazie.

[31] Tiwari RL (2021). A Wnt5a-Cdc42 axis controls aging and rejuvenation of hair-follicle stem cells. Aging (Albany NY).

[32] de Paula DRM (2017). Biological properties of cardiac mesenchymal stem cells in rats with diabetic cardiomyopathy. Life Sci.

[33] Al Hasan M (2021). Type III collagen is required for adipogenesis and actin stress fibre formation in 3T3-L1 preadipocytes. Biomolecules.

[34] Timirci-Kahraman O (2018). A study of short- and long-term mRNA levels of the Retn, Iapp, and Drd5 genes in obese mice induced with high-fat diet. In Vivo.

[35] Zheng J (2021). miR-148a-3p silences the CANX/MHC-I pathway and impairs CD8(+) T cell-mediated immune attack in colorectal cancer. FASEB J.

[36] Zhang M (2021). Targeting the Lnc-OPHN1-5/androgen receptor/hnRNPA1 complex increases Enzalutamide sensitivity to better suppress prostate cancer progression. Cell Death Dis.

[37] HashemiSheikhshabani S (2021). Oleuropein reduces cisplatin resistance in ovarian cancer by targeting apoptotic pathway regulators. Life Sci.

[38] Manzano-Munoz A (2021). MCL-1 inhibition overcomes anti-apoptotic adaptation to targeted therapies in B-cell precursor acute lymphoblastic leukemia. Front Cell Dev Biol.

[39] Yang L (2021). Overexpression of TICRR and PPIF confer poor prognosis in endometrial cancer identified by gene co-expression network analysis. Aging (Albany NY).

[40] Liang C (2021). Construction of adipogenic ceRNA network based on lncRNA expression profile of adipogenic differentiation of human MSC cells. Biochem Genet.

[41] Combs TP (2004). A transgenic mouse with a deletion in the collagenous domain of adiponectin displays elevated circulating adiponectin and improved insulin sensitivity. Endocrinology.

[42] Kusminski CM (2012). MitoNEET-driven alterations in adipocyte mitochondrial activity reveal a crucial adaptive process that preserves insulin sensitivity in obesity. Nat Med.

