# Supplementary material for: Characterization of DNA methylation as well as mico-RNA expression and screening of epigenetic markers in adipogenesis
Source: J Transl Med. 2022 Feb 15;20:93. doi: 10.1186/s12967-022-03295-w (PMC8845261; doi:10.1186/s12967-022-03295-w)


Supplementary Table 1. Gene ontology and KEGG pathway analysis of DEGs associated with aberrant miRNA between early stage and late stage samples.

| **Category** | **Term** | **Count** | **Generatio** | **P value** |
| --- | --- | --- | --- | --- |
| ***Low miRNA targeting up-regulated genes*** | |  |  |  |
| Biological Process | purine-containing compound metabolic process | 13 | 13/95 | 5.16E-07 |
| Biological Process | ribose phosphate metabolic process | 12 | 12/95 | 1.89E-06 |
| Biological Process | purine nucleotide metabolic process | 12 | 12/95 | 2.17E-06 |
| Biological Process | purine ribonucleotide metabolic process | 11 | 11/95 | 6.49E-06 |
| Biological Process | ribonucleotide metabolic process | 11 | 11/95 | 9.54E-06 |
| Cellular Component | intrinsic component of organelle membrane | 9 | 9/96 | 0.000162781 |
| Cellular Component | integral component of organelle membrane | 7 | 7/96 | 0.002371367 |
| Cellular Component | vacuolar membrane | 7 | 7/96 | 0.005385545 |
| Cellular Component | mitochondrial matrix | 7 | 7/96 | 0.009080996 |
| Cellular Component | mitochondrial inner membrane | 7 | 7/96 | 0.01087496 |
| Molecular Function | ubiquitin protein ligase binding | 6 | 6/96 | 0.004409426 |
| Molecular Function | ubiquitin-like protein ligase binding | 6 | 6/96 | 0.005959887 |
| Molecular Function | phosphoric ester hydrolase activity | 6 | 6/96 | 0.01266589 |
| Molecular Function | phosphoprotein phosphatase activity | 5 | 5/96 | 0.002503304 |
| Molecular Function | phosphatase activity | 5 | 5/96 | 0.015003322 |
| KEGG Pathway | Salmonella infection | 6 | 6/49 | 0.003684422 |
| KEGG Pathway | Insulin signaling pathway | 5 | 5/49 | 0.001354963 |
| KEGG Pathway | Parkinson disease | 5 | 5/49 | 0.021674691 |
| KEGG Pathway | Renal cell carcinoma | 4 | 4/49 | 0.000768046 |
| ***High miRNA targeting down-regulated genes*** | |  |  |  |
| Biological Process | regulation of chromosome organization | 8 | 8/62 | 9.09E-05 |
| Biological Process | chromosome segregation | 8 | 8/62 | 0.000163284 |
| Biological Process | nuclear division | 7 | 7/62 | 3.07E-05 |
| Biological Process | condensed chromosome | 7 | 7/62 | 0.000115567 |
| Biological Process | chromosomal region | 7 | 7/62 | 0.000552205 |
| Cellular Component | condensed chromosome kinetochore | 7 | 7/62 | 5.58E-06 |
| Cellular Component | condensed chromosome, centromeric region | 6 | 6/62 | 0.000780057 |
| Cellular Component | kinetochore | 5 | 5/62 | 2.17E-05 |
| Cellular Component | protein kinase regulator activity | 5 | 5/62 | 3.50E-05 |
| Cellular Component | kinase regulator activity | 5 | 5/62 | 7.43E-05 |
| Molecular Function | cadherin binding | 4 | 4/63 | 0.004131437 |
| Molecular Function | small GTPase binding | 4 | 4/63 | 0.006911262 |
| Molecular Function | GTPase binding | 4 | 4/63 | 0.027262903 |
| Molecular Function | Cell cycle | 3 | 3/63 | 0.027616624 |
| Molecular Function | Human T-cell leukemia virus 1 infection | 3 | 3/63 | 0.040900938 |
| KEGG Pathway | MicroRNAs in cancer | 4 | 4/34 | 0.00180966 |
| KEGG Pathway | Oocyte meiosis | 4 | 4/34 | 0.013409031 |
| KEGG Pathway | Spliceosome | 4 | 4/34 | 0.039663022 |
| KEGG Pathway | regulation of chromosome organization | 3 | 3/34 | 0.017205769 |
| KEGG Pathway | chromosome segregation | 3 | 3/34 | 0.023291621 |

Supplementary Table 2. Gene ontology and KEGG pathway analysis of DEGs associated with aberrant DNA methylation between early stage and late stage samples.

| **Category** | **Term** | **Count** | **Generatio** | ***P* value** |
| --- | --- | --- | --- | --- |
| ***Hypomethylation and up-regulated genes*** | | | | |
| Biological Process | small molecule catabolic process | 42 | 8/62 | 9.09E-05 |
| Biological Process | regulation of lipid metabolic process | 41 | 8/62 | 0.000163284 |
| Biological Process | fatty acid metabolic process | 40 | 7/62 | 3.07E-05 |
| Biological Process | regulation of small molecule metabolic process | 39 | 7/62 | 0.000115567 |
| Biological Process | purine-containing compound metabolic process | 39 | 7/62 | 0.000552205 |
| Cellular Component | mitochondrial inner membrane | 57 | 7/62 | 5.58E-06 |
| Cellular Component | mitochondrial matrix | 51 | 6/62 | 0.000780057 |
| Cellular Component | mitochondrial protein-containing complex | 28 | 5/62 | 2.17E-05 |
| Cellular Component | mitochondrial outer membrane | 27 | 5/62 | 3.50E-05 |
| Cellular Component | organelle outer membrane | 27 | 5/62 | 7.43E-05 |
| Molecular Function | tubulin binding | 24 | 4/63 | 0.004131437 |
| Molecular Function | transferase activity, transferring acyl groups | 20 | 4/63 | 0.006911262 |
| Molecular Function | protein-macromolecule adaptor activity | 20 | 4/63 | 0.027262903 |
| Molecular Function | microtubule binding | 19 | 3/63 | 0.027616624 |
| Molecular Function | carboxylic acid binding | 18 | 3/63 | 0.040900938 |
| KEGG Pathway | Alzheimer disease | 27 | 4/34 | 0.00180966 |
| KEGG Pathway | Prion disease | 23 | 4/34 | 0.013409031 |
| KEGG Pathway | Diabetic cardiomyopathy | 22 | 4/34 | 0.039663022 |
| KEGG Pathway | Parkinson disease | 21 | 3/34 | 0.017205769 |
| KEGG Pathway | Chemical carcinogenesis - reactive oxygen species | 19 | 3/34 | 0.023291621 |
| ***Hypermethylation and down-regulated genes*** | | | | |
| Biological Process | extracellular matrix organization | 22 | 22/218 | 1.14E-09 |
| Biological Process | extracellular structure organization | 22 | 22/218 | 1.20E-09 |
| Biological Process | external encapsulating structure organization | 22 | 22/218 | 1.32E-09 |
| Biological Process | skeletal system development | 21 | 21/218 | 2.37E-07 |
| Biological Process | muscle cell differentiation | 17 | 17/218 | 1.13E-06 |
| Cellular Component | collagen-containing extracellular matrix | 21 | 21/227 | 2.59E-08 |
| Cellular Component | focal adhesion | 14 | 14/227 | 0.000386454 |
| Cellular Component | cell-substrate junction | 14 | 14/227 | 0.000456237 |
| Cellular Component | endoplasmic reticulum lumen | 12 | 12/227 | 0.000258184 |
| Cellular Component | basement membrane | 9 | 9/227 | 1.50E-06 |
| Molecular Function | extracellular matrix structural constituent | 14 | 14/224 | 2.28E-08 |
| Molecular Function | growth factor binding | 11 | 11/224 | 9.72E-07 |
| Molecular Function | transmembrane receptor protein kinase activity | 7 | 7/224 | 5.51E-05 |
| Molecular Function | collagen binding | 6 | 6/224 | 0.00018088 |
| Molecular Function | transforming growth factor beta binding | 5 | 5/224 | 9.15E-06 |
| KEGG Pathway | MicroRNAs in cancer | 11 | 11/103 | 0.001876216 |
| KEGG Pathway | PI3K-Akt signaling pathway | 10 | 10/103 | 0.014349458 |
| KEGG Pathway | Focal adhesion | 9 | 9/103 | 0.001004604 |
| KEGG Pathway | Proteoglycans in cancer | 9 | 9/103 | 0.001154425 |
| KEGG Pathway | MAPK signaling pathway | 9 | 9/103 | 0.012337642 |

Supplementary Table 3. DEGs associated with both specific miRNA and DNA methylation CpG sites between early and late stage.

| Gene | DMP | location | chromosome | miRNA |
| --- | --- | --- | --- | --- |
| ***Up-regulated genes affected by both low miRNA and hypomethylation*** | | | | |
| ACACA | cg00276752 | 5'UTR | chr17:35715796-35716627 | hsa-miR-1207-5p |
| ACACA | cg00356511 | 5'UTR | chr17:35715796-35716627 |  |
| ACACA | cg01423843 | TSS200 | chr17:35715796-35716627 |  |
| ACACA | cg01929530 | TSS200 | chr17:35766664-35767335 |  |
| ACACA | cg06899192 | TSS200 | chr17:35766664-35767335 |  |
| ACACA | cg07375836 | 5'UTR | chr17:35715796-35716627 |  |
| ACACA | cg07389276 | 5'UTR | chr17:35715796-35716627 |  |
| ACACA | cg09036203 | 5'UTR | chr17:35715796-35716627 |  |
| ACACA | cg09371063 | 5'UTR | chr17:35715796-35716627 |  |
| ACACA | cg11904960 | 5'UTR | chr17:35715796-35716627 |  |
| ACACA | cg15515895 | 1stExon | chr17:35766664-35767335 |  |
| ACACA | cg15839435 | TSS200 | chr17:35766664-35767335 |  |
| ACACA | cg17783593 | TSS200 | chr17:35715796-35716627 |  |
| ACACA | cg18378490 | TSS200 | chr17:35766664-35767335 |  |
| ACACA | cg18421160 | 5'UTR | chr17:35715796-35716627 |  |
| ACACA | cg19786784 | TSS200 | chr17:35715796-35716627 |  |
| ACACA | cg21401740 | 5'UTR | chr17:35766664-35767335 |  |
| ACACA | cg24455359 | 1stExon | chr17:35715796-35716627 |  |
| ACACA | cg27200190 | 1stExon | chr17:35766664-35767335 |  |
| ALDH2 | cg10449070 | 5'UTR | chr12:112204498-112204979 | hsa-miR-615-3p |
| ALDH2 | cg10887937 | TSS1500 | chr12:112204498-112204979 |  |
| ALDH2 | cg13955512 | 5'UTR | chr12:112204498-112204979 |  |
| ALDH2 | cg18535456 | TSS1500 | chr12:112204498-112204979 |  |
| ALDH2 | cg18780217 | 5'UTR | chr12:112204498-112204979 |  |
| ALDH2 | cg19186356 | TSS200 | chr12:112204498-112204979 |  |
| ALDH2 | cg21470387 | 5'UTR | chr12:112204498-112204979 |  |
| ALDH2 | cg22158248 | TSS1500 | chr12:112204498-112204979 |  |
| ALDH2 | cg24546205 | 1stExon | chr12:112204498-112204979 |  |
| AP1G1 | cg00680808 | 5'UTR | chr16:71841923-71843101 | hsa-miR-513a-5p |
| AP1G1 | cg01140985 | TSS1500 | chr16:71841923-71843101 | hsa-miR-920 |
| AP1G1 | cg02147032 | 5'UTR | chr16:71841923-71843101 |  |
| AP1G1 | cg03047098 | TSS200 | chr16:71841923-71843101 |  |
| AP1G1 | cg03865041 | 5'UTR | chr16:71841923-71843101 |  |
| AP1G1 | cg05535798 | TSS200 | chr16:71841923-71843101 |  |
| AP1G1 | cg06411322 | 5'UTR | chr16:71841923-71843101 |  |
| AP1G1 | cg09931891 | TSS200 | chr16:71841923-71843101 |  |
| AP1G1 | cg10407113 | TSS1500 | chr16:71841923-71843101 |  |
| AP1G1 | cg13701124 | 5'UTR | chr16:71841923-71843101 |  |
| AP1G1 | cg15824968 | 5'UTR | chr16:71841923-71843101 |  |
| AP1G1 | cg16422316 | TSS1500 | chr16:71841923-71843101 |  |
| AP1G1 | cg16666698 | 5'UTR | chr16:71841923-71843101 |  |
| AP1G1 | cg27373657 | TSS200 | chr16:71841923-71843101 |  |
| ARIH1 | cg00343217 | 1stExon | chr15:72766260-72767693 | hsa-miR-1275 |
| ARIH1 | cg03945322 | TSS200 | chr15:72766260-72767693 |  |
| ARIH1 | cg07479864 | TSS200 | chr15:72766260-72767693 |  |
| ARIH1 | cg07488073 | TSS1500 | chr15:72766260-72767693 |  |
| ARIH1 | cg07594478 | TSS200 | chr15:72766260-72767693 |  |
| ARIH1 | cg12251884 | 5'UTR | chr15:72766260-72767693 |  |
| ARIH1 | cg19136717 | 5'UTR | chr15:72766260-72767693 |  |
| ARIH1 | cg20046875 | TSS200 | chr15:72766260-72767693 |  |
| ARIH1 | cg23888240 | TSS1500 | chr15:72766260-72767693 |  |
| ARIH1 | cg25990494 | TSS200 | chr15:72766260-72767693 |  |
| ARIH1 | cg26880891 | 1stExon | chr15:72766260-72767693 |  |
| BLOC1S2 | cg01017197 | TSS1500 | chr10:102046142-102046632 | hsa-miR-595 |
| BLOC1S2 | cg02550027 | TSS1500 | chr10:102046142-102046632 |  |
| BLOC1S2 | cg04774227 | TSS1500 | chr10:102046142-102046632 |  |
| BLOC1S2 | cg07802220 | TSS1500 | chr10:102046142-102046632 |  |
| BLOC1S2 | cg13817732 | 1stExon | chr10:102046142-102046632 |  |
| BLOC1S2 | cg14282612 | TSS1500 | chr10:102046142-102046632 |  |
| BLOC1S2 | cg15177315 | TSS1500 | chr10:102046142-102046632 |  |
| BLOC1S2 | cg15298486 | TSS1500 | chr10:102046142-102046632 |  |
| BLOC1S2 | cg23341182 | TSS1500 | chr10:102046142-102046632 |  |
| BLOC1S2 | cg26610808 | TSS1500 | chr10:102046142-102046632 |  |
| C5orf51 | cg01169772 | TSS1500 | chr5:41904032-41904629 | hsa-miR-513a-5p |
| C5orf51 | cg01194782 | TSS1500 | chr5:41904032-41904629 |  |
| C5orf51 | cg04923388 | 1stExon | chr5:41904032-41904629 |  |
| C5orf51 | cg09176847 | TSS1500 | chr5:41904032-41904629 |  |
| C5orf51 | cg11886014 | 1stExon | chr5:41904032-41904629 |  |
| C5orf51 | cg14671666 | TSS200 | chr5:41904032-41904629 |  |
| C5orf51 | cg15531486 | TSS1500 | chr5:41904032-41904629 | hsa-miR-615-3p |
| C5orf51 | cg16487101 | TSS200 | chr5:41904032-41904629 |  |
| C5orf51 | cg26067897 | TSS200 | chr5:41904032-41904629 |  |
| CANX | cg01329270 | 5'UTR | chr5:179125341-179126340 |  |
| CANX | cg03868400 | 5'UTR | chr5:179125341-179126340 |  |
| CANX | cg04073204 | 3'UTR | chr5:179159467-179160534 |  |
| CANX | cg04652522 | TSS1500 | chr5:179125341-179126340 |  |
| CANX | cg05190557 | TSS1500 | chr5:179125341-179126340 |  |
| CANX | cg07352245 | TSS200 | chr5:179125341-179126340 |  |
| CANX | cg07831432 | TSS1500 | chr5:179125341-179126340 |  |
| CANX | cg08691740 | TSS1500 | chr5:179125341-179126340 |  |
| CANX | cg08716348 | TSS200 | chr5:179125341-179126340 |  |
| CANX | cg09173344 | TSS200 | chr5:179125341-179126340 |  |
| CANX | cg10666364 | TSS200 | chr5:179125341-179126340 |  |
| CANX | cg12865675 | 3'UTR | chr5:179159467-179160534 |  |
| CANX | cg13859478 | 5'UTR | chr5:179125341-179126340 |  |
| CANX | cg14593053 | 5'UTR | chr5:179125341-179126340 |  |
| CANX | cg24689420 | 5'UTR | chr5:179125341-179126340 |  |
| CANX | cg26284905 | 5'UTR | chr5:179125341-179126340 |  |
| EGLN2 | cg03287692 | 1stExon | chr19:41306982-41307313 | hsa-miR-4267 |
| EGLN2 | cg08078058 | 1stExon | chr19:41306982-41307313 |  |
| EGLN2 | cg08080060 | 1stExon | chr19:41306982-41307313 |  |
| EGLN2 | cg08467108 | TSS200 | chr19:41304467-41305050 |  |
| EGLN2 | cg08494502 | TSS1500 | chr19:41304467-41305050 |  |
| EGLN2 | cg10585486 | TSS1500 | chr19:41304467-41305050 |  |
| EGLN2 | cg11298343 | 5'UTR | chr19:41306982-41307313 |  |
| EGLN2 | cg18540299 | 3'UTR | chr19:41316543-41317318 |  |
| EGLN2 | cg22499964 | TSS1500 | chr19:41304467-41305050 |  |
| EGLN2 | cg22671726 | 5'UTR | chr19:41306982-41307313 |  |
| EGLN2 | cg23743927 | TSS200 | chr19:41304467-41305050 |  |
| EGLN2 | cg25923056 | 1stExon | chr19:41306982-41307313 |  |
| EGLN2 | cg26227592 | 3'UTR | chr19:41316543-41317318 |  |
| EGLN2 | cg26338873 | TSS200 | chr19:41304467-41305050 |  |
| EGLN2 | cg27357151 | TSS200 | chr19:41304467-41305050 |  |
| EXOC2 | cg01095518 | 5'UTR | chr6:656409-657155 | hsa-miR-615-3p |
| EXOC2 | cg01870519 | TSS1500 | chr6:692464-693348 |  |
| EXOC2 | cg02375313 | 5'UTR | chr6:656409-657155 |  |
| EXOC2 | cg03107817 | 5'UTR | chr6:692464-693348 |  |
| EXOC2 | cg04789318 | 5'UTR | chr6:656409-657155 |  |
| EXOC2 | cg08352886 | TSS1500 | chr6:692464-693348 |  |
| EXOC2 | cg08460580 | 5'UTR | chr6:692464-693348 |  |
| EXOC2 | cg08754317 | TSS200 | chr6:692464-693348 |  |
| EXOC2 | cg17555940 | 5'UTR | chr6:656409-657155 |  |
| EXOC2 | cg21771171 | 5'UTR | chr6:692464-693348 |  |
| EXOC2 | cg21987077 | 5'UTR | chr6:656409-657155 |  |
| EXOC2 | cg24140573 | TSS200 | chr6:692464-693348 |  |
| EXOC2 | cg25314360 | 5'UTR | chr6:656409-657155 |  |
| EXOC2 | cg27149530 | 5'UTR | chr6:692464-693348 |  |
| EXOC7 | cg00953403 | 5'UTR | chr17:74099738-74100055 | hsa-miR-615-3p |
| EXOC7 | cg02212737 | 1stExon | chr17:74099738-74100055 |  |
| EXOC7 | cg05254689 | TSS1500 | chr17:74099738-74100055 |  |
| EXOC7 | cg08122232 | TSS1500 | chr17:74099738-74100055 |  |
| EXOC7 | cg08642743 | TSS1500 | chr17:74099738-74100055 |  |
| EXOC7 | cg09289278 | 3'UTR | chr17:74070404-74073530 |  |
| EXOC7 | cg10783680 | 5'UTR | chr17:74099738-74100055 |  |
| EXOC7 | cg16628201 | TSS1500 | chr17:74099738-74100055 |  |
| EXOC7 | cg18586095 | TSS1500 | chr17:74099738-74100055 |  |
| EXOC7 | cg19578297 | TSS1500 | chr17:74099738-74100055 |  |
| EXOC7 | cg19757154 | TSS1500 | chr17:74099738-74100055 |  |
| EXOC7 | cg20470722 | TSS1500 | chr17:74099738-74100055 |  |
| EXOC7 | cg21016177 | TSS1500 | chr17:74099738-74100055 |  |
| GLUL | cg00383081 | TSS1500 | chr1:182359697-182361563 | hsa-miR-615-3p |
| GLUL | cg01419479 | 1stExon | chr1:182359697-182361563 |  |
| GLUL | cg01588464 | 5'UTR | chr1:182359697-182361563 |  |
| GLUL | cg03970588 | 1stExon | chr1:182359697-182361563 |  |
| GLUL | cg04017672 | 5'UTR | chr1:182359697-182361563 |  |
| GLUL | cg07386190 | TSS200 | chr1:182359697-182361563 |  |
| GLUL | cg07841877 | 5'UTR | chr1:182359697-182361563 |  |
| GLUL | cg09470059 | TSS200 | chr1:182359697-182361563 |  |
| GLUL | cg12535596 | 5'UTR | chr1:182359697-182361563 |  |
| GLUL | cg13086581 | 1stExon | chr1:182359697-182361563 |  |
| GLUL | cg13180005 | 5'UTR | chr1:182359697-182361563 |  |
| GLUL | cg14851700 | TSS1500 | chr1:182359697-182361563 |  |
| GLUL | cg15114328 | TSS1500 | chr1:182359697-182361563 |  |
| GLUL | cg15389472 | TSS200 | chr1:182359697-182361563 |  |
| GLUL | cg17283022 | TSS200 | chr1:182359697-182361563 |  |
| GLUL | cg17800870 | TSS1500 | chr1:182359697-182361563 |  |
| GLUL | cg17892328 | TSS1500 | chr1:182359697-182361563 |  |
| GLUL | cg20492121 | 1stExon | chr1:182359697-182361563 |  |
| GLUL | cg24868926 | 5'UTR | chr1:182359697-182361563 |  |
| GLUL | cg26135716 | 1stExon | chr1:182359697-182361563 |  |
| GLUL | cg27525037 | TSS200 | chr1:182359697-182361563 |  |
| MACROD2 | cg01552272 | TSS200 | chr20:13975768-13976287 | hsa-miR-486-5p |
| MACROD2 | cg04624110 | TSS200 | chr20:13975768-13976287 |  |
| MACROD2 | cg04716990 | TSS200 | chr20:13975768-13976287 |  |
| MACROD2 | cg06571075 | TSS200 | chr20:13975768-13976287 |  |
| MACROD2 | cg11497924 | TSS1500 | chr20:13975768-13976287 |  |
| MACROD2 | cg18213713 | TSS1500 | chr20:13975768-13976287 |  |
| MACROD2 | cg21856067 | TSS1500 | chr20:13975768-13976287 |  |
| MACROD2 | cg22447508 | TSS1500 | chr20:13975768-13976287 |  |
| MACROD2 | cg23169957 | TSS200 | chr20:13975768-13976287 |  |
| MACROD2 | cg23751724 | 1stExon | chr20:13976700-13977068 |  |
| MACROD2 | cg25557432 | TSS200 | chr20:13975768-13976287 |  |
| MACROD2 | cg26059153 | 1stExon | chr20:13975768-13976287 |  |
| NECAP1 | cg00911551 | TSS200 | chr12:8234689-8235020 | hsa-miR-513a-5p |
| NECAP1 | cg03054141 | TSS200 | chr12:8234689-8235020 |  |
| NECAP1 | cg06401027 | TSS200 | chr12:8234689-8235020 |  |
| NECAP1 | cg06563686 | 1stExon | chr12:8234689-8235020 |  |
| NECAP1 | cg07269271 | TSS200 | chr12:8234689-8235020 |  |
| NECAP1 | cg11138731 | TSS200 | chr12:8234689-8235020 |  |
| NECAP1 | cg11501644 | TSS1500 | chr12:8234689-8235020 |  |
| NECAP1 | cg13443953 | TSS1500 | chr12:8234689-8235020 |  |
| NECAP1 | cg17274400 | 1stExon | chr12:8234689-8235020 |  |
| NECAP1 | cg19255118 | TSS200 | chr12:8234689-8235020 |  |
| NECAP1 | cg22585957 | 1stExon | chr12:8234689-8235020 |  |
| NUCKS1 | cg04873517 | 5'UTR | chr1:205718238-205719345 | hsa-miR-920 |
| NUCKS1 | cg05051117 | TSS1500 | chr1:205718238-205719345 |  |
| NUCKS1 | cg05386151 | 5'UTR | chr1:205718238-205719345 |  |
| NUCKS1 | cg06665535 | TSS200 | chr1:205718238-205719345 |  |
| NUCKS1 | cg09161758 | TSS1500 | chr1:205718238-205719345 |  |
| NUCKS1 | cg09821112 | TSS200 | chr1:205718238-205719345 |  |
| NUCKS1 | cg10021156 | TSS1500 | chr1:205718238-205719345 |  |
| NUCKS1 | cg14239491 | 5'UTR | chr1:205718238-205719345 |  |
| NUCKS1 | cg15006843 | TSS1500 | chr1:205718238-205719345 |  |
| NUCKS1 | cg24606240 | TSS1500 | chr1:205718238-205719345 |  |
| NUCKS1 | cg26894091 | 5'UTR | chr1:205718238-205719345 |  |
| PPIF | cg00357888 | TSS1500 | chr10:81107082-81107488 | hsa-miR-513a-5p |
| PPIF | cg03648155 | TSS1500 | chr10:81107082-81107488 | hsa-miR-615-3p |
| PPIF | cg07441565 | TSS200 | chr10:81107082-81107488 |  |
| PPIF | cg08514558 | TSS1500 | chr10:81107082-81107488 |  |
| PPIF | cg09469691 | TSS200 | chr10:81107082-81107488 |  |
| PPIF | cg11057378 | TSS200 | chr10:81107082-81107488 |  |
| PPIF | cg11858474 | 1stExon | chr10:81107082-81107488 |  |
| PPIF | cg14204211 | TSS200 | chr10:81107082-81107488 |  |
| PPIF | cg16098780 | 1stExon | chr10:81107082-81107488 |  |
| PPIF | cg23585575 | TSS200 | chr10:81107082-81107488 |  |
| PPIF | cg27352063 | TSS200 | chr10:81107082-81107488 |  |
| PRPSAP1 | cg00127591 | TSS200 | chr17:74349151-74350656 | hsa-miR-615-3p |
| PRPSAP1 | cg00549574 | TSS1500 | chr17:74349151-74350656 |  |
| PRPSAP1 | cg00951085 | TSS200 | chr17:74349151-74350656 |  |
| PRPSAP1 | cg01756327 | TSS200 | chr17:74349151-74350656 |  |
| PRPSAP1 | cg02392176 | TSS200 | chr17:74349151-74350656 |  |
| PRPSAP1 | cg06617769 | TSS1500 | chr17:74349151-74350656 |  |
| PRPSAP1 | cg11234111 | TSS200 | chr17:74349151-74350656 |  |
| PRPSAP1 | cg12612336 | TSS1500 | chr17:74349151-74350656 |  |
| PRPSAP1 | cg13949903 | 1stExon | chr17:74349151-74350656 |  |
| PRPSAP1 | cg14816748 | 1stExon | chr17:74349151-74350656 |  |
| PRPSAP1 | cg18322765 | 3'UTR | chr17:74303478-74304428 |  |
| PTPRF | cg00007221 | 5'UTR | chr1:43996724-43997592 | hsa-miR-1275 |
| PTPRF | cg01624937 | 5'UTR | chr1:43996724-43997592 |  |
| PTPRF | cg13233799 | 1stExon | chr1:43996724-43997592 |  |
| PTPRF | cg14172596 | TSS200 | chr1:43996724-43997592 |  |
| PTPRF | cg16570917 | TSS1500 | chr1:43996724-43997592 |  |
| PTPRF | cg17213946 | 5'UTR | chr1:43996724-43997592 |  |
| PTPRF | cg18815355 | TSS1500 | chr1:43996724-43997592 |  |
| PTPRF | cg19824710 | TSS1500 | chr1:43996724-43997592 |  |
| PTPRF | cg21268658 | 5'UTR | chr1:43996724-43997592 |  |
| PTPRF | cg25063733 | 1stExon | chr1:43996724-43997592 |  |
| PTPRF | cg26064481 | 5'UTR | chr1:43996724-43997592 |  |
| SAR1B | cg01948148 | TSS200 | chr5:133968214-133968722 | hsa-miR-1207-5p |
| SAR1B | cg02589828 | 5'UTR | chr5:133968214-133968722 |  |
| SAR1B | cg10441365 | TSS200 | chr5:133968214-133968722 |  |
| SAR1B | cg15385386 | 5'UTR | chr5:133968214-133968722 |  |
| SAR1B | cg15625785 | TSS1500 | chr5:133968214-133968722 |  |
| SAR1B | cg15985126 | TSS1500 | chr5:133968214-133968722 |  |
| SAR1B | cg18260973 | TSS200 | chr5:133968214-133968722 |  |
| SAR1B | cg18318704 | TSS200 | chr5:133968214-133968722 |  |
| SAR1B | cg21544075 | TSS1500 | chr5:133968214-133968722 |  |
| SAR1B | cg21558508 | TSS200 | chr5:133968214-133968722 |  |
| SAR1B | cg25644150 | 5'UTR | chr5:133968214-133968722 |  |
| SAR1B | cg26293310 | TSS200 | chr5:133968214-133968722 |  |
| SH3GLB1 | cg04513669 | TSS1500 | chr1:87169875-87171015 | hsa-miR-3646 |
| SH3GLB1 | cg09799432 | TSS200 | chr1:87169875-87171015 | hsa-miR-767-5p |
| SH3GLB1 | cg10625266 | TSS1500 | chr1:87169875-87171015 |  |
| SH3GLB1 | cg11204830 | TSS1500 | chr1:87169875-87171015 |  |
| SH3GLB1 | cg16116363 | 1stExon | chr1:87169875-87171015 |  |
| SH3GLB1 | cg18172823 | TSS1500 | chr1:87169875-87171015 |  |
| SH3GLB1 | cg22024145 | 5'UTR | chr1:87169875-87171015 |  |
| SH3GLB1 | cg23588844 | TSS200 | chr1:87169875-87171015 |  |
| SH3GLB1 | cg26616378 | TSS1500 | chr1:87169875-87171015 |  |
| SOCS7 | cg01496491 | 1stExon | chr17:36507657-36508939 | hsa-miR-615-3p |
| SOCS7 | cg02741359 | 1stExon | chr17:36507657-36508939 |  |
| SOCS7 | cg02937763 | TSS1500 | chr17:36507657-36508939 |  |
| SOCS7 | cg07535751 | TSS200 | chr17:36507657-36508939 |  |
| SOCS7 | cg11819707 | TSS200 | chr17:36507657-36508939 |  |
| SOCS7 | cg12247306 | TSS1500 | chr17:36507657-36508939 |  |
| SOCS7 | cg15251212 | 1stExon | chr17:36507657-36508939 |  |
| SOCS7 | cg16571354 | 1stExon | chr17:36507657-36508939 |  |
| SOCS7 | cg27527736 | TSS1500 | chr17:36507657-36508939 |  |
| TMEM126B | cg00287711 | TSS1500 | chr11:85339343-85339629 | hsa-miR-615-3p |
| TMEM126B | cg03746685 | 5'UTR | chr11:85339343-85339629 |  |
| TMEM126B | cg06779329 | 5'UTR | chr11:85339343-85339629 |  |
| TMEM126B | cg07739727 | 1stExon | chr11:85339343-85339629 |  |
| TMEM126B | cg10845902 | TSS1500 | chr11:85339343-85339629 |  |
| TMEM126B | cg11799072 | TSS200 | chr11:85339343-85339629 |  |
| TMEM126B | cg12830327 | TSS200 | chr11:85339343-85339629 |  |
| TMEM126B | cg12984656 | TSS1500 | chr11:85339343-85339629 |  |
| TMEM126B | cg13711609 | TSS1500 | chr11:85339343-85339629 |  |
| TMEM126B | cg16292382 | TSS200 | chr11:85339343-85339629 |  |
| TMEM126B | cg17340796 | TSS200 | chr11:85339343-85339629 |  |
| TMEM126B | cg17597195 | TSS200 | chr11:85339343-85339629 |  |
| TMEM126B | cg18142026 | TSS1500 | chr11:85339343-85339629 |  |
| TMEM126B | cg20275558 | TSS1500 | chr11:85339343-85339629 |  |
| TMEM126B | cg20588946 | TSS1500 | chr11:85339343-85339629 |  |
| TMEM126B | cg21787323 | TSS1500 | chr11:85339343-85339629 |  |
| TMEM126B | cg25820704 | TSS1500 | chr11:85339343-85339629 |  |
| TPST2 | cg00149397 | TSS200 | chr22:26985516-26986264 | hsa-miR-615-3p |
| TPST2 | cg02370373 | 5'UTR | chr22:26985516-26986264 |  |
| TPST2 | cg05873732 | TSS200 | chr22:26985516-26986264 |  |
| TPST2 | cg07255019 | 5'UTR | chr22:26985516-26986264 |  |
| TPST2 | cg09243400 | TSS200 | chr22:26985516-26986264 |  |
| TPST2 | cg09856467 | 5'UTR | chr22:26936996-26937672 |  |
| TPST2 | cg13657092 | TSS1500 | chr22:26985516-26986264 |  |
| TPST2 | cg15333769 | 5'UTR | chr22:26985516-26986264 |  |
| TPST2 | cg19708343 | TSS200 | chr22:26985516-26986264 |  |
| TPST2 | cg23365832 | 5'UTR | chr22:26985516-26986264 |  |
| ZBTB20 | cg03692305 | 5'UTR | chr3:114865436-114867124 | hsa-miR-513a-5p |
| ZBTB20 | cg04403629 | TSS1500 | chr3:114865436-114867124 |  |
| ZBTB20 | cg04809093 | 5'UTR | chr3:114865436-114867124 |  |
| ZBTB20 | cg04917686 | TSS1500 | chr3:114865436-114867124 |  |
| ZBTB20 | cg07144984 | 5'UTR | chr3:114865436-114867124 |  |
| ZBTB20 | cg07365274 | TSS200 | chr3:114865436-114867124 |  |
| ZBTB20 | cg11130461 | 5'UTR | chr3:114865436-114867124 |  |
| ZBTB20 | cg12763900 | TSS200 | chr3:114865436-114867124 |  |
| ZBTB20 | cg15091879 | TSS200 | chr3:114865436-114867124 |  |
| ZBTB20 | cg16263825 | TSS1500 | chr3:114865436-114867124 |  |
| ZBTB20 | cg16515820 | TSS1500 | chr3:114865436-114867124 |  |
| ZBTB20 | cg18660891 | TSS1500 | chr3:114865436-114867124 |  |
| ZBTB20 | cg20784525 | TSS1500 | chr3:114865436-114867124 |  |
| ZBTB20 | cg25364469 | 5'UTR | chr3:114069725-114070658 |  |
| ZBTB20 | cg26152597 | 5'UTR | chr3:114865436-114867124 |  |
| ZBTB20 | cg26539949 | 5'UTR | chr3:114069725-114070658 |  |
| Gene | DMP | location | chromosome | miRNA |
| ***down-regulated genes affected by both high miRNA and hypermethylation*** | | | | |
| ATP8B2 | cg00581482 | 5'UTR | chr1:154298205-154298544 | hsa-miR-423-3p |
| ATP8B2 | cg06811467 | TSS1500 | chr1:154300975-154301528 |  |
| ATP8B2 | cg06824583 | TSS1500 | chr1:154298205-154298544 |  |
| ATP8B2 | cg07244253 | TSS1500 | chr1:154298205-154298544 |  |
| ATP8B2 | cg08190044 | 5'UTR | chr1:154298205-154298544 |  |
| ATP8B2 | cg10212705 | TSS200 | chr1:154298205-154298544 |  |
| ATP8B2 | cg12644885 | TSS200 | chr1:154298205-154298544 |  |
| ATP8B2 | cg15026150 | TSS200 | chr1:154298205-154298544 |  |
| ATP8B2 | cg16013543 | TSS200 | chr1:154300975-154301528 |  |
| ATP8B2 | cg17094927 | 5'UTR | chr1:154298205-154298544 |  |
| ATP8B2 | cg18813777 | TSS1500 | chr1:154298205-154298544 |  |
| ATP8B2 | cg19760410 | TSS200 | chr1:154300975-154301528 |  |
| ATP8B2 | cg20744437 | TSS1500 | chr1:154298205-154298544 |  |
| ATP8B2 | cg21379008 | TSS1500 | chr1:154298205-154298544 |  |
| ATP8B2 | cg23233141 | TSS200 | chr1:154298205-154298544 |  |
| ATP8B2 | cg23924887 | TSS1500 | chr1:154298205-154298544 |  |
| ATP8B2 | cg24040570 | TSS1500 | chr1:154298205-154298544 |  |
| ATP8B2 | cg24296484 | 5'UTR | chr1:154298205-154298544 |  |
| BAIAP2 | cg01066451 | 3'UTR | chr17:79084336-79084561 | hsa-miR-455-3p |
| BAIAP2 | cg01158079 | 3'UTR | chr17:79086321-79086523 |  |
| BAIAP2 | cg04242349 | 3'UTR | chr17:79084336-79084561 |  |
| BAIAP2 | cg06174144 | 3'UTR | chr17:79084336-79084561 |  |
| BAIAP2 | cg08039592 | 3'UTR | chr17:79086321-79086523 |  |
| BAIAP2 | cg09859805 | 3'UTR | chr17:79086321-79086523 |  |
| BAIAP2 | cg10637425 | TSS1500 | chr17:79008929-79009762 |  |
| BAIAP2 | cg15010854 | TSS1500 | chr17:79008929-79009762 |  |
| BAIAP2 | cg17341933 | TSS1500 | chr17:79008929-79009762 |  |
| BAIAP2 | cg23210075 | 3'UTR | chr17:79093076-79096677 |  |
| BAIAP2 | cg23532322 | 3'UTR | chr17:79086321-79086523 |  |
| BAIAP2 | cg27343663 | 3'UTR | chr17:79084336-79084561 |  |
| BAIAP2 | cg27551910 | 3'UTR | chr17:79084336-79084561 |  |
| CALU | cg01174859 | TSS200 | chr7:128379114-128379697 | hsa-miR-107 |
| CALU | cg03534322 | 5'UTR | chr7:128379114-128379697 | hsa-miR-4289 |
| CALU | cg04434696 | TSS200 | chr7:128379114-128379697 |  |
| CALU | cg04774374 | TSS1500 | chr7:128379114-128379697 |  |
| CALU | cg05647526 | 5'UTR | chr7:128379114-128379697 |  |
| CALU | cg08307369 | 1stExon | chr7:128379114-128379697 |  |
| CALU | cg08742095 | 5'UTR | chr7:128379114-128379697 |  |
| CALU | cg09436892 | TSS1500 | chr7:128379114-128379697 |  |
| CALU | cg10313005 | 5'UTR | chr7:128379114-128379697 |  |
| CALU | cg10365046 | TSS200 | chr7:128379114-128379697 |  |
| CALU | cg19402173 | 1stExon | chr7:128379114-128379697 |  |
| CALU | cg19426362 | TSS200 | chr7:128379114-128379697 |  |
| CALU | cg23475963 | TSS200 | chr7:128379114-128379697 |  |
| CALU | cg27031506 | TSS1500 | chr7:128379114-128379697 |  |
| CALU | cg27045724 | TSS200 | chr7:128379114-128379697 |  |
| DFFB | cg00905725 | TSS1500 | chr1:3773187-3774465 | hsa-miR-421 |
| DFFB | cg03808734 | TSS1500 | chr1:3773187-3774465 |  |
| DFFB | cg04835615 | TSS200 | chr1:3773187-3774465 |  |
| DFFB | cg08459627 | 5'UTR | chr1:3773187-3774465 |  |
| DFFB | cg08889585 | TSS200 | chr1:3773187-3774465 |  |
| DFFB | cg10444080 | TSS200 | chr1:3773187-3774465 |  |
| DFFB | cg12679050 | TSS1500 | chr1:3773187-3774465 |  |
| DFFB | cg14213620 | 5'UTR | chr1:3773187-3774465 |  |
| DFFB | cg15525503 | TSS1500 | chr1:3773187-3774465 |  |
| DFFB | cg16135451 | TSS1500 | chr1:3773187-3774465 |  |
| DFFB | cg18237277 | 1stExon | chr1:3773187-3774465 |  |
| DFFB | cg20822256 | 5'UTR | chr1:3773187-3774465 |  |
| DFFB | cg24546680 | TSS200 | chr1:3773187-3774465 |  |
| FKBP14 | cg02350474 | TSS1500 | chr7:30067762-30068600 | hsa-miR-3142 |
| FKBP14 | cg03105133 | TSS200 | chr7:30066419-30066685 |  |
| FKBP14 | cg03361067 | TSS1500 | chr7:30066419-30066685 |  |
| FKBP14 | cg11601662 | 1stExon | chr7:30066419-30066685 |  |
| FKBP14 | cg11802692 | TSS200 | chr7:30066419-30066685 |  |
| FKBP14 | cg13527910 | TSS1500 | chr7:30066419-30066685 |  |
| FKBP14 | cg14688751 | TSS200 | chr7:30066419-30066685 |  |
| FKBP14 | cg16743785 | TSS200 | chr7:30066419-30066685 |  |
| FKBP14 | cg18851071 | TSS1500 | chr7:30066419-30066685 |  |
| FKBP14 | cg18898185 | TSS200 | chr7:30066419-30066685 |  |
| HNRNPA1 | cg02049941 | TSS1500 | chr12:54673322-54673550 | hsa-miR-324-5p |
| HNRNPA1 | cg03689456 | TSS1500 | chr12:54673322-54673550 |  |
| HNRNPA1 | cg06399839 | TSS1500 | chr12:54673814-54674988 |  |
| HNRNPA1 | cg09060496 | TSS1500 | chr12:54673814-54674988 |  |
| HNRNPA1 | cg09158487 | TSS1500 | chr12:54673814-54674988 |  |
| HNRNPA1 | cg09653879 | TSS1500 | chr12:54673322-54673550 |  |
| HNRNPA1 | cg11338003 | TSS1500 | chr12:54673814-54674988 |  |
| HNRNPA1 | cg12263485 | TSS1500 | chr12:54673814-54674988 |  |
| HNRNPA1 | cg14017196 | TSS1500 | chr12:54673814-54674988 |  |
| HNRNPA1 | cg14394617 | 1stExon | chr12:54673814-54674988 |  |
| HNRNPA1 | cg15249411 | TSS1500 | chr12:54673322-54673550 |  |
| HNRNPA1 | cg20927027 | 1stExon | chr12:54673814-54674988 |  |
| HOXC4 | cg00040312 | 5'UTR | chr12:54424610-54425173 | hsa-miR-125a-5p |
| HOXC4 | cg00187380 | 5'UTR | chr12:54427025-54428709 |  |
| HOXC4 | cg00218447 | 5'UTR | chr12:54427025-54428709 |  |
| HOXC4 | cg00243574 | 5'UTR | chr12:54411709-54412131 |  |
| HOXC4 | cg00506343 | 5'UTR | chr12:54423427-54423712 |  |
| HOXC4 | cg00567703 | 5'UTR | chr12:54412990-54413346 |  |
| HOXC4 | cg00576279 | 5'UTR | chr12:54427025-54428709 |  |
| HOXC4 | cg01153660 | TSS200 | chr12:54411709-54412131 |  |
| HOXC4 | cg01473837 | TSS1500 | chr12:54408426-54408713 |  |
| HOXC4 | cg01524853 | 1stExon | chr12:54447744-54448091 |  |
| HOXC4 | cg01579535 | 5'UTR | chr12:54447744-54448091 |  |
| HOXC4 | cg01683044 | TSS200 | chr12:54411709-54412131 |  |
| HOXC4 | cg01704924 | 5'UTR | chr12:54447744-54448091 |  |
| HOXC4 | cg01946191 | 5'UTR | chr12:54445876-54446113 |  |
| HOXC4 | cg02017718 | 5'UTR | chr12:54440642-54441543 |  |
| HOXC4 | cg02264990 | 5'UTR | chr12:54447744-54448091 |  |
| HOXC4 | cg02470595 | 5'UTR | chr12:54427025-54428709 |  |
| HOXC4 | cg02491017 | TSS1500 | chr12:54411709-54412131 |  |
| HOXC4 | cg02721000 | 5'UTR | chr12:54440642-54441543 |  |
| HOXC4 | cg03144714 | 5'UTR | chr12:54427025-54428709 |  |
| HOXC4 | cg03207151 | 5'UTR | chr12:54440642-54441543 |  |
| HOXC4 | cg03255182 | 5'UTR | chr12:54423427-54423712 |  |
| HOXC4 | cg03305955 | 5'UTR | chr12:54412990-54413346 |  |
| HOXC4 | cg03416628 | 5'UTR | chr12:54447744-54448091 |  |
| HOXC4 | cg03668274 | 5'UTR | chr12:54427025-54428709 |  |
| HOXC4 | cg03892356 | 5'UTR | chr12:54423427-54423712 |  |
| HOXC4 | cg03923561 | 5'UTR | chr12:54447744-54448091 |  |
| HOXC4 | cg04105511 | TSS1500 | chr12:54408426-54408713 |  |
| HOXC4 | cg04576672 | 5'UTR | chr12:54423427-54423712 |  |
| HOXC4 | cg04704531 | TSS1500 | chr12:54408426-54408713 |  |
| HOXC4 | cg04794183 | TSS200 | chr12:54411709-54412131 |  |
| HOXC4 | cg05296643 | TSS200 | chr12:54411709-54412131 |  |
| HOXC4 | cg05349837 | 5'UTR | chr12:54427025-54428709 |  |
| HOXC4 | cg05408649 | 5'UTR | chr12:54445876-54446113 |  |
| HOXC4 | cg05416450 | 5'UTR | chr12:54411709-54412131 |  |
| HOXC4 | cg05477933 | 5'UTR | chr12:54427025-54428709 |  |
| HOXC4 | cg05982757 | 5'UTR | chr12:54427025-54428709 |  |
| HOXC4 | cg05987823 | 5'UTR | chr12:54427025-54428709 |  |
| HOXC4 | cg05992786 | 5'UTR | chr12:54447744-54448091 |  |
| HOXC4 | cg06714180 | TSS1500 | chr12:54408426-54408713 |  |
| HOXC4 | cg07080050 | 5'UTR | chr12:54412990-54413346 |  |
| HOXC4 | cg07266404 | 5'UTR | chr12:54447744-54448091 |  |
| HOXC4 | cg07545037 | 5'UTR | chr12:54424610-54425173 |  |
| HOXC4 | cg07687119 | 5'UTR | chr12:54423427-54423712 |  |
| HOXC4 | cg07915976 | 5'UTR | chr12:54412990-54413346 |  |
| HOXC4 | cg08106887 | 5'UTR | chr12:54412990-54413346 |  |
| HOXC4 | cg08465346 | 5'UTR | chr12:54440642-54441543 |  |
| HOXC4 | cg08712054 | 5'UTR | chr12:54447744-54448091 |  |
| HOXC4 | cg09481972 | 5'UTR | chr12:54411709-54412131 |  |
| HOXC4 | cg09720701 | 5'UTR | chr12:54447744-54448091 |  |
| HOXC4 | cg10005224 | 5'UTR | chr12:54424610-54425173 |  |
| HOXC4 | cg10366797 | 5'UTR | chr12:54440642-54441543 |  |
| HOXC4 | cg10593493 | 5'UTR | chr12:54411709-54412131 |  |
| HOXC4 | cg10918927 | 5'UTR | chr12:54411709-54412131 |  |
| HOXC4 | cg11585893 | 5'UTR | chr12:54447744-54448091 |  |
| HOXC4 | cg11594833 | 5'UTR | chr12:54445876-54446113 |  |
| HOXC4 | cg11746813 | 1stExon | chr12:54447744-54448091 |  |
| HOXC4 | cg11941633 | 5'UTR | chr12:54423427-54423712 |  |
| HOXC4 | cg12232783 | 5'UTR | chr12:54423427-54423712 |  |
| HOXC4 | cg12905836 | TSS1500 | chr12:54411709-54412131 |  |
| HOXC4 | cg13726459 | TSS1500 | chr12:54408426-54408713 |  |
| HOXC4 | cg13826247 | 5'UTR | chr12:54424610-54425173 |  |
| HOXC4 | cg14108840 | TSS1500 | chr12:54411709-54412131 |  |
| HOXC4 | cg14644523 | 5'UTR | chr12:54427025-54428709 |  |
| HOXC4 | cg15233062 | 5'UTR | chr12:54447744-54448091 |  |
| HOXC4 | cg15244786 | 5'UTR | chr12:54447744-54448091 |  |
| HOXC4 | cg15611151 | 5'UTR | chr12:54412990-54413346 |  |
| HOXC4 | cg15660418 | TSS1500 | chr12:54408426-54408713 |  |
| HOXC4 | cg15700739 | 5'UTR | chr12:54427025-54428709 |  |
| HOXC4 | cg15718289 | 5'UTR | chr12:54411709-54412131 |  |
| HOXC4 | cg15772924 | 5'UTR | chr12:54411709-54412131 |  |
| HOXC4 | cg15817960 | TSS1500 | chr12:54408426-54408713 |  |
| HOXC4 | cg15834355 | 5'UTR | chr12:54440642-54441543 |  |
| HOXC4 | cg15894722 | 5'UTR | chr12:54447744-54448091 |  |
| HOXC4 | cg16765387 | 5'UTR | chr12:54411709-54412131 |  |
| HOXC4 | cg16937769 | TSS1500 | chr12:54408426-54408713 |  |
| HOXC4 | cg16983211 | 5'UTR | chr12:54427025-54428709 |  |
| HOXC4 | cg17026220 | TSS200 | chr12:54411709-54412131 |  |
| HOXC4 | cg17031478 | 5'UTR | chr12:54427025-54428709 |  |
| HOXC4 | cg17827660 | 5'UTR | chr12:54411709-54412131 |  |
| HOXC4 | cg18040878 | TSS1500 | chr12:54408426-54408713 |  |
| HOXC4 | cg18054172 | 5'UTR | chr12:54424610-54425173 |  |
| HOXC4 | cg18695839 | 5'UTR | chr12:54447744-54448091 |  |
| HOXC4 | cg18843682 | 5'UTR | chr12:54424610-54425173 |  |
| HOXC4 | cg18922524 | 5'UTR | chr12:54447744-54448091 |  |
| HOXC4 | cg19058685 | 5'UTR | chr12:54440642-54441543 |  |
| HOXC4 | cg19164987 | 5'UTR | chr12:54412990-54413346 |  |
| HOXC4 | cg19182597 | 5'UTR | chr12:54427025-54428709 |  |
| HOXC4 | cg19186380 | 5'UTR | chr12:54427025-54428709 |  |
| HOXC4 | cg19696083 | 5'UTR | chr12:54440642-54441543 |  |
| HOXC4 | cg19892287 | 5'UTR | chr12:54412990-54413346 |  |
| HOXC4 | cg20381985 | 5'UTR | chr12:54411709-54412131 |  |
| HOXC4 | cg21041775 | 5'UTR | chr12:54427025-54428709 |  |
| HOXC4 | cg21216477 | 5'UTR | chr12:54440642-54441543 |  |
| HOXC4 | cg21476000 | 5'UTR | chr12:54427025-54428709 |  |
| HOXC4 | cg21487207 | TSS200 | chr12:54411709-54412131 |  |
| HOXC4 | cg21493516 | 5'UTR | chr12:54445876-54446113 |  |
| HOXC4 | cg21582112 | 5'UTR | chr12:54423427-54423712 |  |
| HOXC4 | cg21703606 | 5'UTR | chr12:54427025-54428709 |  |
| HOXC4 | cg22151644 | 5'UTR | chr12:54445876-54446113 |  |
| HOXC4 | cg22198132 | TSS1500 | chr12:54411709-54412131 |  |
| HOXC4 | cg22358580 | 5'UTR | chr12:54427025-54428709 |  |
| HOXC4 | cg22370252 | 5'UTR | chr12:54447744-54448091 |  |
| HOXC4 | cg22378817 | 5'UTR | chr12:54423427-54423712 |  |
| HOXC4 | cg22525581 | 5'UTR | chr12:54440642-54441543 |  |
| HOXC4 | cg22621272 | TSS1500 | chr12:54408426-54408713 |  |
| HOXC4 | cg22747076 | 1stExon | chr12:54447744-54448091 |  |
| HOXC4 | cg23047434 | TSS200 | chr12:54411709-54412131 |  |
| HOXC4 | cg23618344 | 5'UTR | chr12:54423427-54423712 |  |
| HOXC4 | cg23697546 | 5'UTR | chr12:54424610-54425173 |  |
| HOXC4 | cg24735790 | 5'UTR | chr12:54427025-54428709 |  |
| HOXC4 | cg24896860 | TSS200 | chr12:54411709-54412131 |  |
| HOXC4 | cg25122233 | 5'UTR | chr12:54412990-54413346 |  |
| HOXC4 | cg25975485 | 5'UTR | chr12:54447744-54448091 |  |
| HOXC4 | cg26019295 | TSS1500 | chr12:54408426-54408713 |  |
| HOXC4 | cg26035702 | 5'UTR | chr12:54411709-54412131 |  |
| HOXC4 | cg26162108 | TSS1500 | chr12:54411709-54412131 |  |
| HOXC4 | cg26201952 | 5'UTR | chr12:54447744-54448091 |  |
| HOXC4 | cg27002522 | 5'UTR | chr12:54427025-54428709 |  |
| HOXC4 | cg27008363 | 3'UTR | chr12:54447744-54448091 |  |
| HOXC4 | cg27138204 | 5'UTR | chr12:54445876-54446113 |  |
| HOXC4 | cg27441225 | 5'UTR | chr12:54423427-54423712 |  |
| MCL1 | cg02961109 | TSS1500 | chr1:150551328-150552353 | hsa-miR-320b |
| MCL1 | cg03756514 | TSS200 | chr1:150551328-150552353 | hsa-miR-320c |
| MCL1 | cg04228104 | 1stExon | chr1:150551328-150552353 | hsa-miR-320d |
| MCL1 | cg06217312 | TSS1500 | chr1:150551328-150552353 | hsa-miR-339-3p |
| MCL1 | cg07561848 | 1stExon | chr1:150551328-150552353 |  |
| MCL1 | cg09726469 | 1stExon | chr1:150551328-150552353 |  |
| MCL1 | cg09804220 | 1stExon | chr1:150551328-150552353 |  |
| MCL1 | cg10858523 | 1stExon | chr1:150551328-150552353 |  |
| MCL1 | cg13175981 | TSS1500 | chr1:150551328-150552353 |  |
| MCL1 | cg16431914 | 1stExon | chr1:150551328-150552353 |  |
| MCL1 | cg17217603 | 1stExon | chr1:150551328-150552353 |  |
| MCL1 | cg17724175 | TSS1500 | chr1:150551328-150552353 |  |
| MCL1 | cg18016565 | TSS1500 | chr1:150551328-150552353 |  |
| MCL1 | cg18397157 | 1stExon | chr1:150551328-150552353 |  |
| MCL1 | cg22649068 | TSS1500 | chr1:150551328-150552353 |  |
| MCL1 | cg26118326 | 3'UTR | chr1:150551328-150552353 |  |
| NDRG1 | cg00799984 | 5'UTR | chr8:134308328-134310145 | hsa-miR-4284 |
| NDRG1 | cg01828328 | TSS1500 | chr8:134308328-134310145 |  |
| NDRG1 | cg05994672 | 1stExon | chr8:134308328-134310145 |  |
| NDRG1 | cg07062933 | TSS1500 | chr8:134308328-134310145 |  |
| NDRG1 | cg07477160 | 1stExon | chr8:134308328-134310145 |  |
| NDRG1 | cg08691775 | TSS1500 | chr8:134308328-134310145 |  |
| NDRG1 | cg09102409 | 5'UTR | chr8:134308328-134310145 |  |
| NDRG1 | cg16001384 | 5'UTR | chr8:134308328-134310145 |  |
| NDRG1 | cg17129188 | 5'UTR | chr8:134308328-134310145 |  |
| NDRG1 | cg17365845 | TSS200 | chr8:134308328-134310145 |  |
| NDRG1 | cg18745406 | TSS1500 | chr8:134308328-134310145 |  |
| NDRG1 | cg18942213 | 1stExon | chr8:134308328-134310145 |  |
| NDRG1 | cg20100745 | 5'UTR | chr8:134308328-134310145 |  |
| NDRG1 | cg25232510 | 5'UTR | chr8:134308328-134310145 |  |
| OSMR | cg02390103 | 5'UTR | chr5:38845502-38846476 | hsa-miR-4284 |
| OSMR | cg03138091 | 5'UTR | chr5:38845502-38846476 |  |
| OSMR | cg05485663 | 5'UTR | chr5:38845502-38846476 |  |
| OSMR | cg05955210 | 5'UTR | chr5:38845502-38846476 |  |
| OSMR | cg11126762 | 5'UTR | chr5:38845502-38846476 |  |
| OSMR | cg15599832 | TSS1500 | chr5:38845502-38846476 |  |
| OSMR | cg17528648 | 5'UTR | chr5:38845502-38846476 |  |
| OSMR | cg19609242 | TSS1500 | chr5:38845502-38846476 |  |
| OSMR | cg22473846 | 5'UTR | chr5:38845502-38846476 |  |
| OSMR | cg23516451 | TSS1500 | chr5:38845502-38846476 |  |
| OSMR | cg26475085 | TSS1500 | chr5:38845502-38846476 |  |
| POFUT2 | cg00500729 | TSS1500 | chr21:46707629-46708751 | hsa-miR-455-3p |
| POFUT2 | cg01267908 | 3'UTR | chr21:46686907-46687121 |  |
| POFUT2 | cg02159808 | TSS200 | chr21:46707629-46708751 |  |
| POFUT2 | cg04097543 | 3'UTR | chr21:46686907-46687121 |  |
| POFUT2 | cg04657831 | TSS200 | chr21:46707629-46708751 |  |
| POFUT2 | cg05732300 | TSS1500 | chr21:46707629-46708751 |  |
| POFUT2 | cg06758583 | 3'UTR | chr21:46685115-46685379 |  |
| POFUT2 | cg06929414 | TSS1500 | chr21:46707629-46708751 |  |
| POFUT2 | cg08041279 | 3'UTR | chr21:46685115-46685379 |  |
| POFUT2 | cg12352984 | 3'UTR | chr21:46685115-46685379 |  |
| POFUT2 | cg12443444 | TSS200 | chr21:46707629-46708751 |  |
| POFUT2 | cg12954385 | 3'UTR | chr21:46686907-46687121 |  |
| POFUT2 | cg13826709 | TSS1500 | chr21:46707629-46708751 |  |
| POFUT2 | cg14247588 | 3'UTR | chr21:46685115-46685379 |  |
| POFUT2 | cg19498844 | TSS200 | chr21:46707629-46708751 |  |
| POFUT2 | cg20607513 | TSS1500 | chr21:46707629-46708751 |  |
| POFUT2 | cg20896738 | TSS200 | chr21:46707629-46708751 |  |
| POFUT2 | cg21885868 | TSS1500 | chr21:46706691-46707049 |  |
| POFUT2 | cg23515696 | TSS200 | chr21:46707629-46708751 |  |
| POFUT2 | cg26108594 | TSS1500 | chr21:46707629-46708751 |  |
| POFUT2 | cg27210852 | 3'UTR | chr21:46685115-46685379 |  |
| SYT7 | cg00009053 | 3'UTR | chr11:61283864-61284147 | hsa-miR-455-3p |
| SYT7 | cg02514519 | TSS1500 | chr11:61347808-61349038 |  |
| SYT7 | cg06405186 | 3'UTR | chr11:61283864-61284147 |  |
| SYT7 | cg08752135 | 3'UTR | chr11:61283864-61284147 |  |
| SYT7 | cg10442157 | 3'UTR | chr11:61283864-61284147 |  |
| SYT7 | cg12693641 | TSS1500 | chr11:61347808-61349038 |  |
| SYT7 | cg14572111 | TSS1500 | chr11:61347808-61349038 |  |
| SYT7 | cg14851471 | TSS1500 | chr11:61347808-61349038 |  |
| SYT7 | cg16965393 | TSS1500 | chr11:61347808-61349038 |  |
| SYT7 | cg17683593 | 3'UTR | chr11:61283864-61284147 |  |
| SYT7 | cg25994096 | 1stExon | chr11:61347808-61349038 |  |
| SYT7 | cg27165920 | 3'UTR | chr11:61283864-61284147 |  |
| TMEM43 | cg00661202 | 1stExon | chr3:14165717-14166999 | hsa-miR-320b |
| TMEM43 | cg01638809 | 1stExon | chr3:14165717-14166999 | hsa-miR-320c |
| TMEM43 | cg08843513 | TSS1500 | chr3:14165717-14166999 | hsa-miR-455-3p |
| TMEM43 | cg10608842 | 1stExon | chr3:14165717-14166999 |  |
| TMEM43 | cg14143241 | 1stExon | chr3:14165717-14166999 |  |
| TMEM43 | cg14375111 | TSS1500 | chr3:14165717-14166999 |  |
| TMEM43 | cg14919823 | 1stExon | chr3:14165717-14166999 |  |
| TMEM43 | cg21666675 | TSS1500 | chr3:14165717-14166999 |  |
| TMEM43 | cg23507024 | 1stExon | chr3:14165717-14166999 |  |
| TMEM43 | cg27109131 | TSS1500 | chr3:14165717-14166999 |  |
| VCAN | cg00328900 | 5'UTR | chr5:82768387-82769268 | hsa-miR-107 |
| VCAN | cg00567749 | 5'UTR | chr5:82768387-82769268 |  |
| VCAN | cg02371408 | TSS1500 | chr5:82768387-82769268 |  |
| VCAN | cg02551029 | TSS200 | chr5:82768387-82769268 |  |
| VCAN | cg04525496 | 1stExon | chr5:82768387-82769268 |  |
| VCAN | cg05176349 | 1stExon | chr5:82768387-82769268 |  |
| VCAN | cg07478641 | 5'UTR | chr5:82768387-82769268 |  |
| VCAN | cg07891434 | 5'UTR | chr5:82770148-82770599 |  |
| VCAN | cg11958643 | 5'UTR | chr5:82770148-82770599 |  |
| VCAN | cg14997132 | 5'UTR | chr5:82770148-82770599 |  |
| VCAN | cg15114672 | TSS1500 | chr5:82768387-82769268 |  |
| VCAN | cg15336997 | 5'UTR | chr5:82770148-82770599 |  |
| VCAN | cg16000227 | TSS1500 | chr5:82768387-82769268 |  |
| VCAN | cg16431436 | 5'UTR | chr5:82768387-82769268 |  |
| VCAN | cg17771652 | 5'UTR | chr5:82768387-82769268 |  |
| VCAN | cg17839359 | 5'UTR | chr5:82768387-82769268 |  |
| VCAN | cg19540689 | TSS200 | chr5:82768387-82769268 |  |
| VCAN | cg19814134 | 5'UTR | chr5:82768387-82769268 |  |
| VCAN | cg21594702 | 5'UTR | chr5:82770148-82770599 |  |
| VCAN | cg21810188 | TSS200 | chr5:82768387-82769268 |  |
| VCAN | cg23936023 | 5'UTR | chr5:82768387-82769268 |  |
| VCAN | cg25660010 | 5'UTR | chr5:82770148-82770599 |  |
| ZC3H4 | cg01264379 | TSS200 | chr19:47615253-47617101 | hsa-miR-423-3p |
| ZC3H4 | cg03288340 | 5'UTR | chr19:47615253-47617101 |  |
| ZC3H4 | cg06189986 | 5'UTR | chr19:47615253-47617101 |  |
| ZC3H4 | cg06627077 | TSS200 | chr19:47615253-47617101 |  |
| ZC3H4 | cg09137630 | TSS1500 | chr19:47615253-47617101 |  |
| ZC3H4 | cg12451679 | TSS200 | chr19:47615253-47617101 |  |
| ZC3H4 | cg18489475 | TSS1500 | chr19:47615253-47617101 |  |
| ZC3H4 | cg23931836 | 5'UTR | chr19:47615253-47617101 |  |
| ZC3H4 | cg24181914 | TSS1500 | chr19:47615253-47617101 |  |
| ZC3H4 | cg24904303 | TSS200 | chr19:47615253-47617101 |  |
| ZC3H4 | cg26306214 | TSS200 | chr19:47615253-47617101 |  |

Figure S1 Transcription factors predicted for hub genes HNRNPA1,PPIF and MCL1（2 transcription factors per gene）.


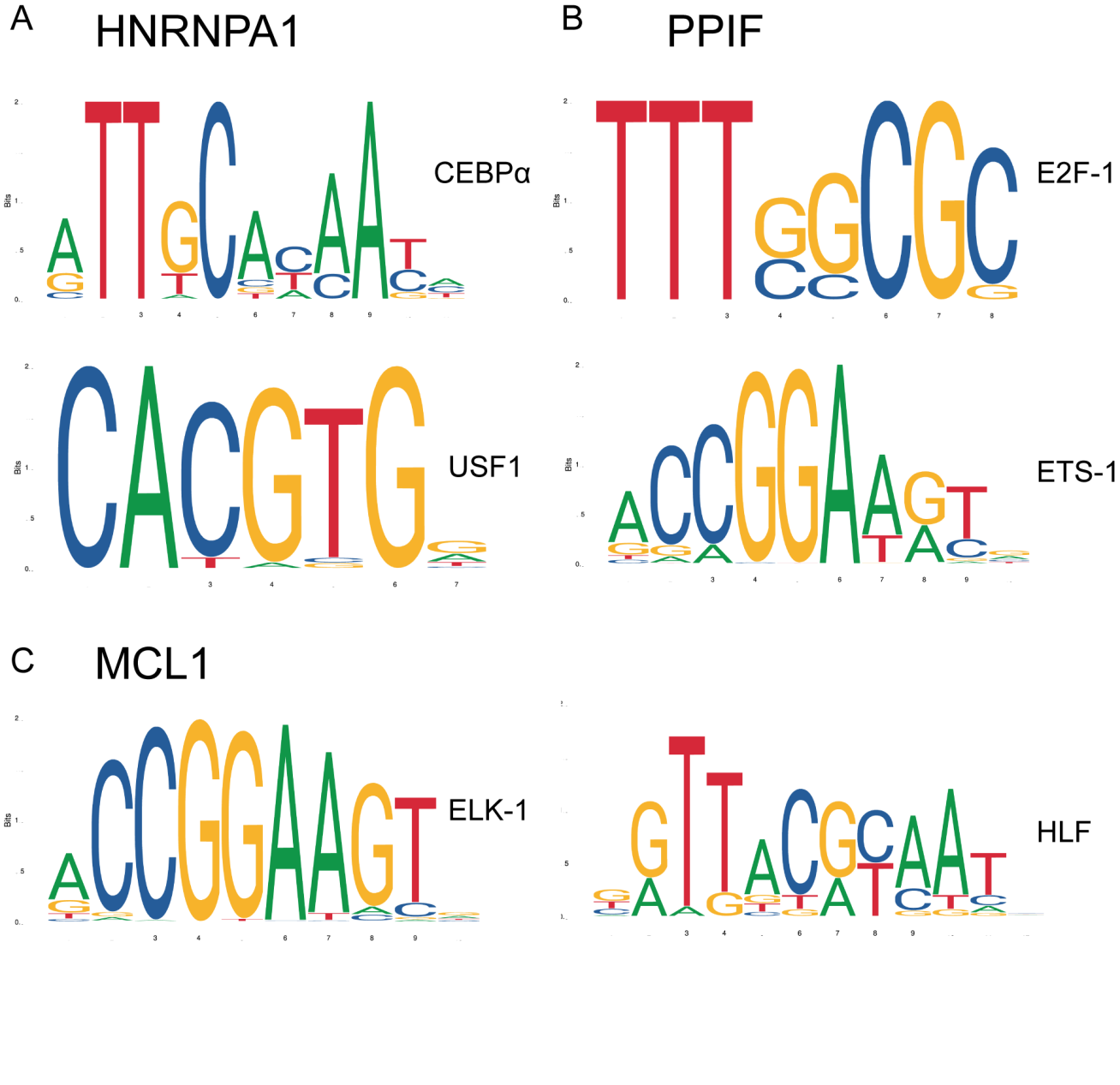

Supplement: Supplementary file 1 — Additional file 1: Table S1. Gene ontology and KEGG pathway analysis of DEGs associated with aberrant miRNA between early stage and late stage samples. Table S2. Gene ontology and KEGG pathway analysis of DEGs associated with aberrant DNA methylation between early stage and late stage samples. Table S3. DEGs associated with both specific miRNA and DNA methylation CpG sites between early and late stage. Figure S1. Transcription factors predicted for hub genes HNRNPA1,PPIF and MCL1 (2 transcription factors per gene. [file 12967_2022_3295_MOESM1_ESM.docx]
